# Sustainable and Direct Upcycling of Waste Graphite Anodes via Deep Eutectic Solvents

**DOI:** 10.1002/advs.202506637

**Published:** 2025-08-18

**Authors:** Xue Liu, Shaoqing Liu, Junhan Pu, Rong Zeng, Tao Wang, Jiashen Meng, Jean‐Jacques Gaumet, Wen Luo, Jianwen Liu

**Affiliations:** ^1^ College of New Energy and Electrical Engineering and College of Chemistry and Chemical Engineering & Ministry of Education Key Laboratory for Green Preparation and Application of Functional Materials Hubei University Wuhan 430062 P. R. China; ^2^ School of Physics and Mechanics Wuhan University of Technology Wuhan 430070 P. R. China; ^3^ State Key Laboratory of Advanced Technology for Materials Synthesis and Processing School of Materials Science and Engineering Wuhan University of Technology Wuhan 430070 P. R. China; ^4^ Laboratoire de Chimie et Physique: Approche Multi‐échelles des Milieux Complexes (LCP‐A2MC) Institut Jean Barriol Université de Lorraine Metz 57070 France

**Keywords:** deep eutectic solvents, phosphate‐rich interfacial film, sustainable and direct upcycling, waste graphite

## Abstract

Lithium‐ion batteries (LIBs) have flourished in power and energy storage, followed by the waste batteries that are pouring into the market. For waste graphite anode, how to deal with high efficiency, high economic efficiency, and low environmental pollution has become a huge challenge. In the work, a deep eutectic solvent (DES), with a low melting point, low cost, and natural environmental protection, is applied as a green reagent to realize the sustainable and direct upcycling of waste graphite. Substances in DES existing in the form of ions and charged P‐containing groups are more likely to attack defect‐rich graphite and realize in situ phosphorus doping, and doped phosphorus participates in the construction of Li_3_PO_4_‐rich solid electrolyte interphase (SEI). Due to the reconstruction of phosphate‐rich interfacial film, the capacity of regenerated graphite maintains as high as 365 mAh g^−1^ at 0.5C with a capacity retention rate of 95.5%, which is much higher than that of waste graphite and even commercial graphite. In addition, the low melting point of DES makes the regeneration temperature significantly lower, so that the strategy reduces the CO_2_ emissions of energy consumption. More importantly, the environment and economy have been optimized, which is conducive to its large‐scale promotion in industry.

## Graphical abstract

A charged particle attacks the vacancy to realize the in‐situ P‐doping model. Cl− anions chelate with charged metal ions for the purpose of impurity removal, and meanwhile the cations bind to the electrons on the vacancy surface to neutralize the electrons. The negatively charged P‐rich group attacks the positively charged vacancy to achieve the purpose of P‐doping.

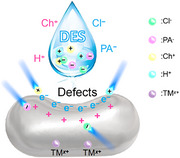

## 1 Introduction

With the increasing demand for lithium‐ion batteries (LIBs) in energy storage, power, and mobile electronic devices, spent batteries have also followed in our field of vision.^[^ ^1^ ^]^ Generally, researchers and companies attach much importance to the recovery of waste metals from the cathodes, such as nickel, cobalt, manganese, etc., however, they always ignore the re‐utilization of waste graphite (W‐G) from the anodes.^[^ ^2^ ^]^ Although the value of graphite is lower than that of valuable metals, graphite production needs to consume huge energy and economic costs, especially in the process of graphitization. Therefore, if the waste graphite in the LIBs is directly discarded, it will cause a huge waste of resources and environmental pollution.^[^ ^3^ ^]^ In line with the purpose of low carbon and low cost, researchers and enterprises are gradually recognizing the potential value of waste graphite.

The scrap mechanism of graphite anode in the LIBs mainly includes structural damage, phase transformation, generation of dead lithium, and damage of the solid electrolyte interface (SEI) films, etc.^[^ ^4^ ^]^ First, the repeated lithiation and de‐lithiation of Li^+^ during the battery cycles bring strong friction and static power between the layers of graphite. This directly leads to the increase of graphite layer spacing, graphite layer stripping, graphitized grain fracture, and the enhancement of anisotropy in the internal structure. Second, the destroyed graphite layer slips along the diagonal direction of the six‐ring carbon structure, resulting in a phase transformation from 2H to 3R phase, which has a low lithium storage capacity.^[^ ^5^ ^]^ Third, the lithium and other metals can escape from the bondage of cathode electrolyte interface (CEI) films during the battery cycles, to be embedded in graphite. Due to the strong electropositivity and metal abundance, some carbon atoms will be removed, further leading to the formation of defects. The destruction of the internal structure eventually causes the diffusion path to be disturbed, resulting in an increase in internal resistance. Additionally, the SEI film on the graphite surface is damaged, and the electrolyte consumption is also the reason for the scrap of graphite.^[^ ^6^ ^]^

Currently, the recycling and re‐utilization methods of waste graphite mainly include high temperature graphitization, hydrometallurgical regeneration and restoration, and the extension of high‐value graphite derivatives etc. For example, Tour et al. employed joule heating to treat the surface SEI film, and then used hydrochloric acid leaching to regenerate waste graphite.^[^ ^7^ ^]^ Wang et al. proposed a method to regenerate and coat waste graphite using asphalt.^[^ ^8^ ^]^ Zhang et al. applied a layer of polymethyl methacrylate to coat on the scrap graphite surface and to reduce the consumption of lithium ions during the SEI film formation.^[^ ^9^ ^]^ Yang et al. used various carbons, such as sucrose, starch, and glucose, to coat on the graphite surface, thus forming the amorphous carbon coatings.^[^ ^10^ ^]^ Based on the above, the high temperature graphitization means impurity removal and graphitization under the high temperature conditions of 2500–3000 °C, which increases the cost of the regeneration process and carbon emissions owing to the tremendous energy consumption.^[^ ^11^ ^]^ By comparison, the hydrometallurgical regeneration is usually uses various acids to dissolve the metals in waste graphite and then uses other carbon sources to repair and regenerate the graphite surface.

In recent years, deep eutectic solvents (DESs) have been developed and favored by researchers as new green solvents with the advantages of low toxicity, low volatility, low corrosion, and environmental friendliness etc.^[^ ^12^ ^]^ Generally, DESs are formed by the combination of hydrogen donor and hydrogen acceptor reagents. The donor and the acceptor form DESs by hydrogen bonding at room temperature, and the generation of hydrogen bonds reduces the lattice energy, which in turn lowers the melting point. Currently, DESs have been gradually applied in the field of spent cathode recovery, for example, choline chloride (ChCl)‐urea,^[^ ^13^ ^]^ ChCl‐p‐toluenesulfonic acid for spent LiCoO_2_,^[^ ^14^ ^]^ ChCl‐oxalic acid,^[^ ^15^ ^]^ ChCl‐ascorbic acid for spent LiNi*_x_* Co*_y_* Mn*_z_* O_2_,^[^ ^16^ ^]^ etc. However, DESs are mostly used for hydrometallurgical leaching rather than direct regeneration; the cathode structures are always destroyed due to their acidity and coordination.

For waste graphite, it is usually embedded with many metal impurities, the surface SEI film is seriously damaged, and the structure itself is also seriously flawed. In view of these drawbacks, the strong coordination ability of DES can effectively remove residual impurities. In addition, the charged ion state functional group of DES can attack the structure defects to form element doping to achieve optimization and upcycling. In our work, phytic acid (PA) is selected as the hydrogen donor reagent with charged phosphorus‐rich groups. ChCl acts as the hydrogen acceptor, in which the strong coordination ability of chloride ions can dissolve excessive metals embedded in graphite. In addition, PA and ChCl are widely present in living organisms and have little impact on the environment. To sum up, the DES upcycling for waste graphite offers several advantages: 1) low energy consumption, 2) short process flows, and 3) natural and environmental protection, making it suitable for industrial production.

## 2 Results and Discussion

### 2.1 Original Properties of DESs

**Figure 1:**
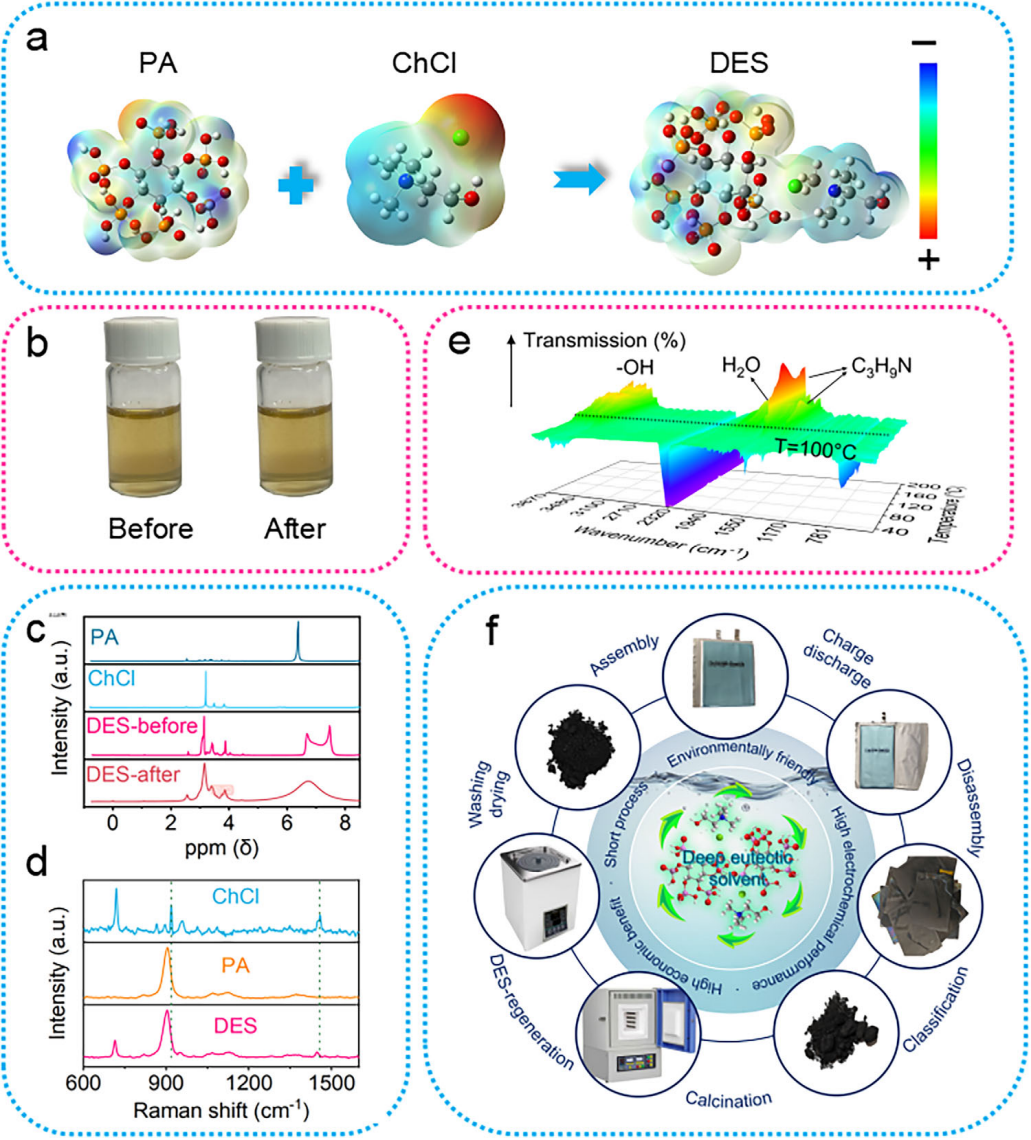
The physicochemical properties of DES. a) Molecular electrostatic potential energy surface illustration of the formation process of DES (Grey: C, White: H, Red: O, Orange: P, Blue: N, Green: Cl). b) The optical photographs of DES before and after regeneration. c) The NMR spectra of PA, ChCl, DES before and after used. d) The Raman spectra of ChCl, PA, and DES. e) The TG‐FTIR spectra of DES with the temperature changes. f) Flow diagram of sustainable and direct upcycling of waste graphite via deep eutectic solvents.

Deep eutectic solvents (DESs), usually composed of two parts hydrogen donor reagent and hydrogen acceptor reagent, are much rich in tanglesome charged anions and cations.^[^ ^17^ ^]^ Among them, the hydrogen donor reagents are generally organic acids or alcohols containing active hydrogen, and the hydrogen acceptor reagents are mostly quaternary ammonium salts, in which the nitrogen (N) atom can accept the active hydrogen in the hydrogen donor reagents. In our work, ChCl is selected as the typical hydrogen acceptor reagent and PA as the hydrogen donor reagent to form DES. As illustrated in **Figure** **1**a, the electrostatic potential of the whole DES system is more uniform due to hydrogen bonds, Lewis acid‐base interaction, and van der Waals force, etc. The ChCl molecular can accept the active hydrogen from PA, while PA molecular with charged phosphorus(P)‐rich groups can realize the direct upcycling for waste graphite via abundant P doping. Simultaneously, the acidity (H^+^) and the coordination (Cl^−^) can achieve efficient removal of metal impurities in waste graphite. Therefore, the as‐formed DES has a low melting point, high P concentration, strong coordination ability. In Figure 1b, it can be observed that the DES is a transparent light‐yellow liquid, and the regenerated DES is still a transparent light‐yellow liquid, and the color of DES after regeneration is slightly darker, only due to the iron (Fe) dissolution. To determine the specific content of iron ions, an Inductively Coupled Plasma (ICP) test is conducted on the leaching solution, and the results are displayed in Figure S1 (Supporting Information). The content of iron ions in the extracted DES is 3514.0 mg mL^−1^. After the calculation, it is approximately the same as the iron impurity content present in graphite. In addition, the remaining dead lithium in the graphite is also leached out, and the content of lithium ions in the leached DES solution is 28.5 mg mL^−1^.

In the NMR spectra of DES in Figure 1c, a new nuclear magnetic peak appears at *δ* = 7.5 ppm, obviously. This phenomenon can be ascribed to the formation of hydrogen bonds between the active hydrogen in PA and ChCl, which reduces the density of H electron cloud on the methyl group adjacent to N and shifts the chemical shift to a higher level. Besides, no new peaks appear or disappear, revealing that only hydrogen bonds are formed during the formation of DES, and no chemical changes occur. Similarly, no new peaks appear or disappear in the post‐used DES, indicating that the structure of DES is not destroyed in the regeneration process, and DES has good stability.^[^ ^18^ ^]^ The same conclusion can also be proved by Raman spectroscopy in Figure 1d, and no new Raman peaks appear in DES. However, the N─CH_3_ (1450 cm^−1^) and CH_3_ (960 cm^−1^) peaks of ChCl shift to a lower angle, which is because N atom, as a hydrogen bond acceptor, forms hydrogen bonds with PA. These hydrogen bonds decrease the binding force of functional groups adjacent to N, thus causing the stretching vibration to move to a low wavenumber.

To explore the optimal reaction temperature, DES mixed with waste graphite was analyzed by TG‐FTIR. The characterization results were supported by Beijing Zhongkebaice Technology Service Co., Ltd. In Figure 1e and Figure S2 (Supporting Information), no new FTIR peaks appear before 100 °C, confirming that DES is stable before 100 °C. However, new FTIR peaks appear at 1450, 1500, and 3500 cm^−1^ as the temperature rises, which originate from the decomposition product trimethylamine from ChCl. New peaks at high wavenumbers over 1600 cm^−1^ are derived from the volatilization of water.^[^ ^19^ ^]^ Therefore, 80 °C is chosen as the optimal reaction condition, which is much lower than the high temperature of graphitization (2500–3000 °C), and lower than the required temperature for carbonized modified graphite (500–700 °C). In our work, the waste graphite is roasted at 600 °C for 2 h, the residual binder and diaphragm are removed, and then the pre‐treated graphite is directly regenerated by DES at 80 °C for 10 h, and the upcycled graphite (DES‐G) will be obtained, which is demonstrated in Figure 1f. Compared with other regeneration methods, this technology greatly reduces the process time and energy consumption, which has bright prospects and is especially suitable for being promoted in the regeneration field of waste graphite.

### 2.2 Structural Properties of Regenerated Graphite

**Figure 2:**
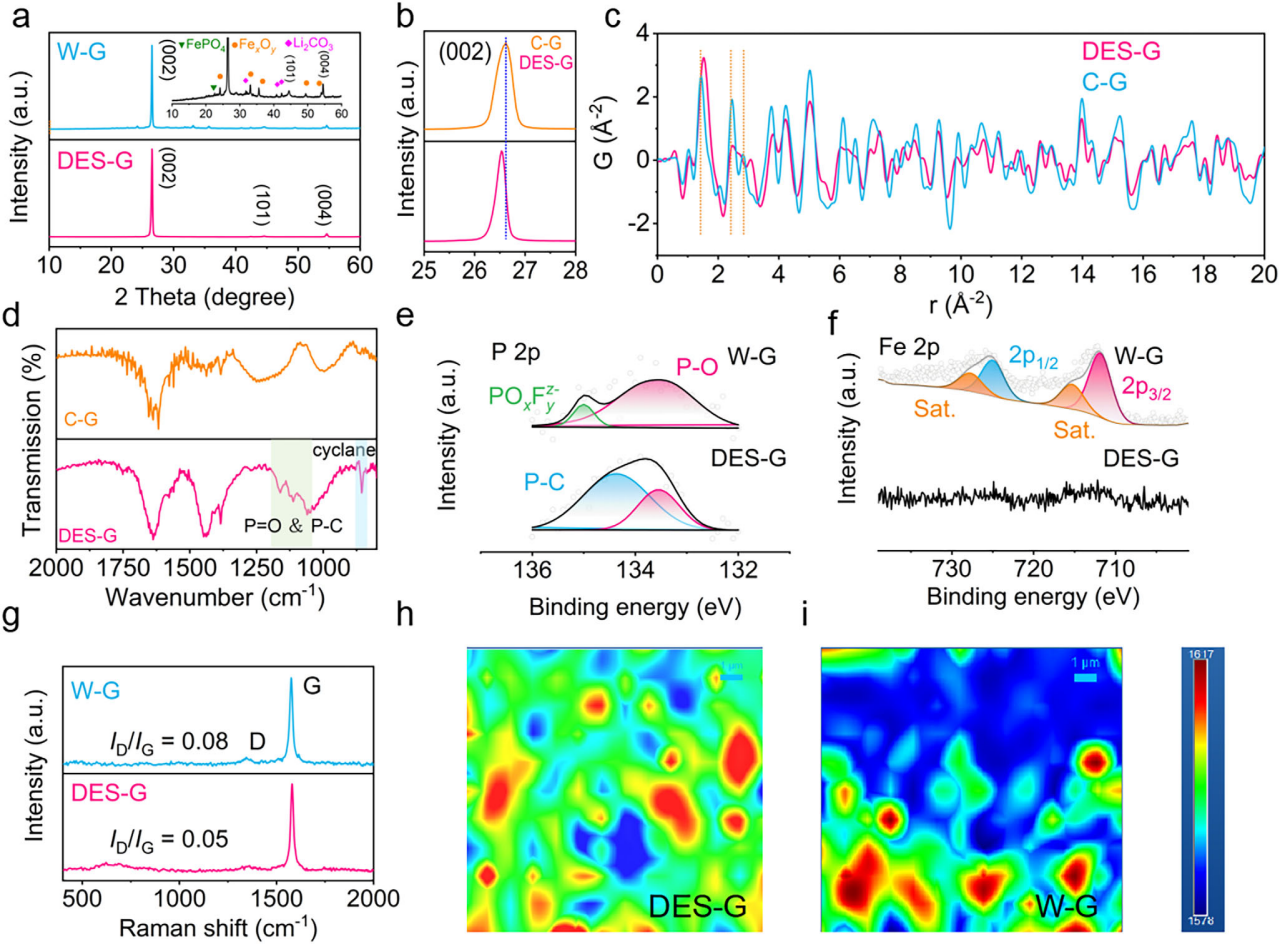
Structural properties of regenerated graphite. a) The XRD patterns of W‐G and DES‐G. b) The (002) peak XRD patterns of C‐G and DES‐G. c) Pair distribution function of DES‐G and C‐G from 0 to 20 Å. d) The FTIR spectra of C‐G and DES‐G. The e) P 2p and f) Fe 2p XPS spectra of W‐G and DES‐G. g) The point scanning Raman spectra of W‐G and DES‐G. The Raman mapping spectra of h) DES‐G and (h) W‐G.

In **Figure** **2**a, many impurity peaks can be obviously observed in the XRD patterns of W‐G, belonging to Fe*_x_* O*_y_*, FePO_4_, Li_2_CO_3,_ and CuO, etc. During the battery cycles, the lithiation and de‐lithiation of Li^+^ has been constantly processed in graphite. When part of the lithium cannot be removed, it will be left in the graphite to form dead lithium, and eventually forming impurities in graphite. The presence of Fe element is due to the damaged CEI film on the cathode surface during the battery cycles, resulting in the inability to constraint the Fe, which is dissolved in the electrolyte and migrates to the anode, thereby embedding in the graphite as impurities. The presence of aluminum and copper is mainly caused by current collector corrosion. In contrast, DES regenerated graphite (DES‐G) after regeneration does not contain any other impurities; only (002) (101) (004) three characteristic peaks of graphite are detected. In Figure 2b, on the other hand, DES‐G has a lower (002) peak Angle than commercial graphite (C‐G). Because phosphorus doping makes the graphite layer spacing slightly larger. More importantly, the most direct and valid evidence is provided by pair distribution function (PDF) in Figure 2c, which reveals the distance between carbon and carbon in the graphite. The shift in peak position indicates that the C─C bond length is different. The distance between the carbon and the three nearest carbons is 1.42, 2.42, and 2.84 Å in C‐G, which is consistent with literature reports and theoretical results.^[^ ^20^ ^]^ In contrast, the PDF peaks of DES‐G shift toward higher values. The distance from carbon to its nearest neighbor is 1.45 Å, which is slightly farther than C‐G. In graphite, carbon is a sp^2^ hybrid structure. Due to the introduction of the P element into the six‐membered ring structure of carbon, the original six‐membered ring structure is destroyed, and the C─C bond becomes longer. However, the shape of the PDF spectra of DES‐G and C‐G is roughly the same, indicating that carbon is still in the same six‐membered ring structure as graphite. The structure of DES‐G is ordered on a long‐term scale, which belongs to the graphite structure. However, on a short‐term scale, there is an increase in bond length caused by the introduction of P elements. Similar evidence can also be provided by FTIR spectra in Figure 2d and Figure S3 (Supporting Information). The peak at 2468 cm^−1^ in W‐G belongs to the vibration of C═O owing to the presence of Li_2_CO_3_. In the regenerated DES‐G, the C═O peak has completely disappeared, and many functional group characteristic peaks corresponding to P‐doped PO_4_, and P‐C appear at 1060 cm^−1^, 1120 cm^−1^, respectively. In addition, there is a skeleton vibration peak of cycloalkane at 860 cm^−1^. This shows that phosphorus replaces carbon and destroys the original benzene ring, proving that phosphorus is introduced into graphite. From the XPS spectra in Figure 2e,f and Figure S3 (Supporting Information), the characteristic peaks of impurity Fe in W‐G exist in the Fe2p spectra mainly in the form of +3 valence, while the presence of Fe is not found in DES‐G. In the P2p spectra, the P element exists in the form of P─O and PO*_x_* F*_y_^z^* ^−^ in W‐G, due to the phosphorus residues from the electrolyte and SEI film, respectively. In contrast, in DES‐G, P exists mainly as the P─C and P─O bonds, confirming again that phosphorus‐doped graphite is successfully regenerated. In the C1s spectra, the C─F and C═O bonds from PVDF and electrolyte residues in W‐G are significantly weakened or disappear after DES regeneration. In the O1s spectra, it can be observed that the O content of DES‐G is significantly lower than that of W‐G, indicating that the oxidized graphite has been effectively repaired.^[^ ^21^ ^]^ In Figure 2g–i, the point scanning and mapping results of the Raman spectra of W‐G and DES‐G are presented. The D and G peaks in the Raman spectra are related to the degree of graphitization of the graphite. Peak D represents defective carbon, and peak G represents regular carbon. The *I*_D_/*I*_G_ value of DES‐G (0.05) is less than that of W‐G (0.08), indicating that the degree of graphitization of graphite after regeneration is improved. An additional characteristic peak at 622 cm^−1^ attributed to the C─P bond emerges, proving that P is successfully doped into the graphite. The more visualized results are contained in the mapping data. The data of DES‐G is more uniform, while the data of W‐G is uneven, indicating that the graphitization of the regenerated material is more uniform.

**Figure 3:**
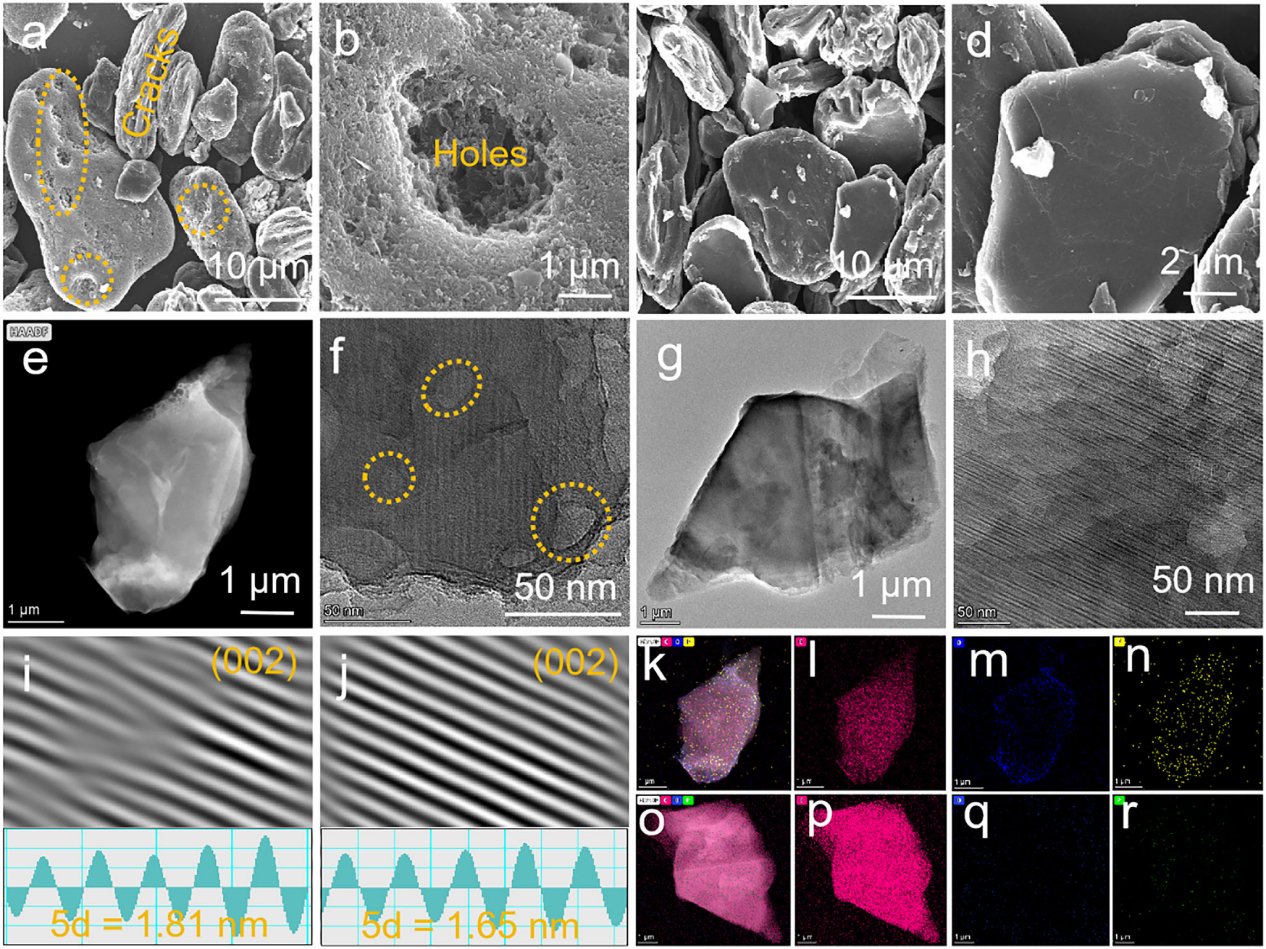
Particle and morphology observations. SEM images of a,b) W‐G and c,d) DES‐G after DES regeneration. TEM images of e,f) W‐G and g,h) DES‐G after D4ES regeneration. The lattice fringes and FFT and IFFT diagrams of i) W‐G and j) DES‐G. The element distributions including C, O, F, and P in k‐n) W‐G and o‐r) DES‐G (Pink: C, Blue: O, Yellow: F, Green: P).

In the SEM images in **Figure** **3**a–d, the surface of W‐G is very rough with many holes and cracks. By comparison, the surface of DES‐G is much smooth, confirming that the graphite is perfectly regenerated. Furtherly, in the TEM images in Figure 3e–h, many holes are also found in W‐G and while only uniform lattice fringes in DES‐G are observed. At the edge of DES‐G, the obvious layered structure can be observed, which belongs to the unique graphite. Meanwhile, in Figure 3i,j, lattice fringes with a width of 0.33 nm are found in DES‐G, corresponding to the (002) crystal face of graphite. It is narrower than the lattice fringe of waste graphite (0.36 nm), due to the successful removal of Li and transition metals embedded in the graphite. This result can also be verified by the XRD patterns, which reveal that the (002) peak shifts to the high angle after the DES regeneration, proving that the lattice spacing decreases.^[^ ^22^ ^]^ In Figure S5 (Supporting Information), two distinct rings belonging to the (002) and (101) crystal faces of graphite emerge in selected area electron diffraction images. And there are no other impurity rings, further proving that the graphite has been successfully regenerated. From the SEM and TEM element information in Figure 3k–r and Figure S6 (Supporting Information), a large amount of F and O distribute in W‐G besides C, while the element distribution of DES‐G mainly contains C, significantly‐reduced O, and obviously‐increased P.

### 2.3 Electrochemical Properties of W‐G, C‐G, and DES‐G

**Figure 4:**
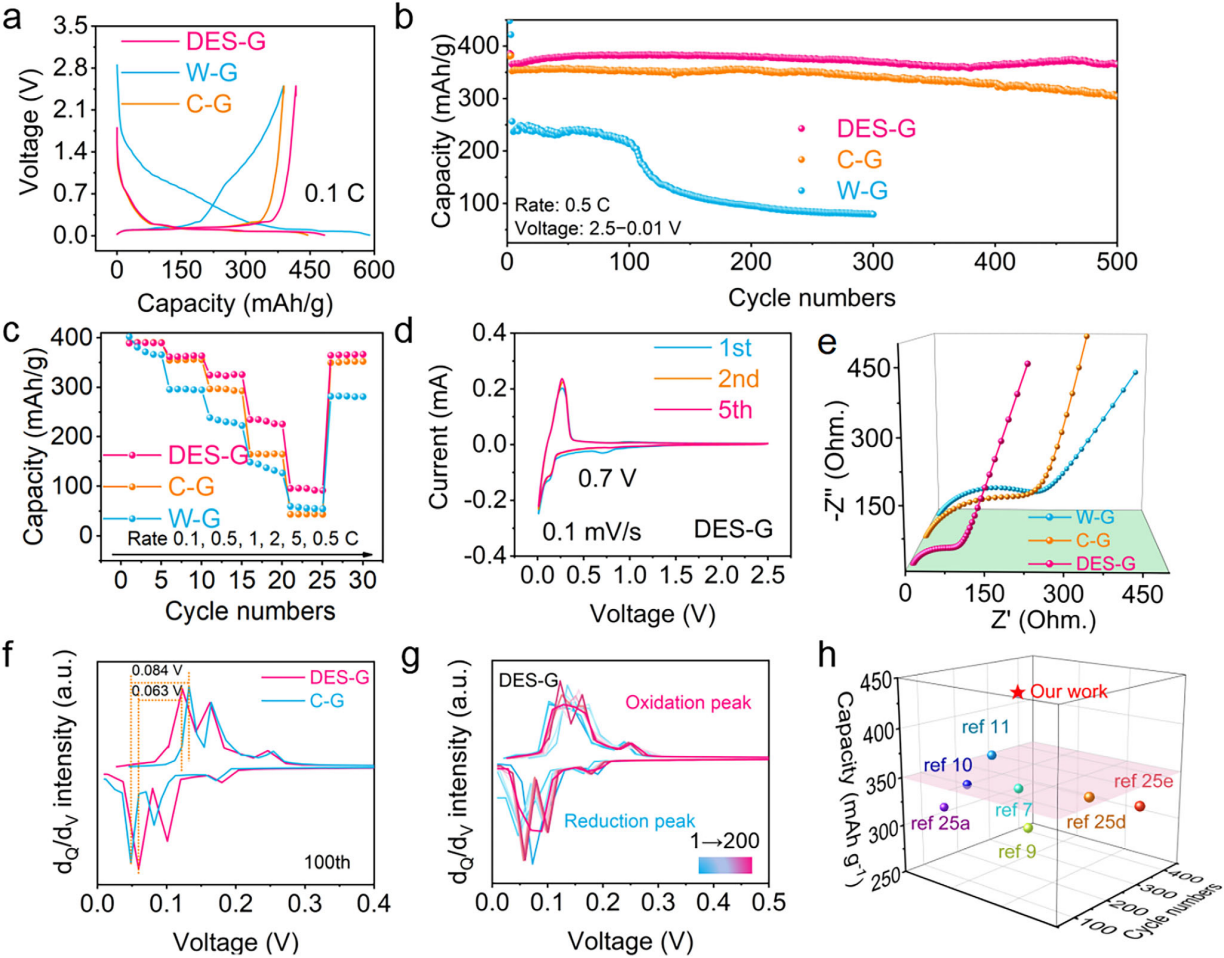
Electrochemical performance of graphite. a) Initial charge and discharge curves at 0.1 C, b) cycling performance, and c) rate performance of DES‐G, W‐G, and C‐G. CV curves of d) DES‐G. e) The electrochemical impedance of DES‐G, W‐G, and C‐G. f) The 100th cycle d*_Q_*/d*_V_* of DES‐G and C‐G curves. g) d*_Q_*/d*_V_* curves of DES‐G and C‐G during 200 cycles. h) Comparison of battery performance between our work and existing reported literatures, and the pink plane shows the electrochemical performance of commercial graphite.

**Figure** **4**a–c displays the comparison of electrochemical properties of commercial graphite and graphite before and after regeneration. In Figure 4a, the DES‐G delivers an initial specific charge capacity as high as 411.1 mAh g^−1^ at 0.1C (1C = 375 mAh g^−1^), which is much higher than that of W‐G (296.1 mAh g^−1^), and even commercial graphite (388.6 mAh g^−1^). This increased capacity is mainly ascribed to the presence of P doping on the surface of DES‐G, thus largely improving the lithium storage performance of graphite. It is worth mentioning that a very large slope emerges in the initial discharge curve of W‐G, which is not the characteristic discharge platform of graphite, but the capacity consumption from impurity elements in graphite and graphite edge defects during the film formation process, confirming that W‐G has been seriously polluted and damaged. In Figure 4b of cycle performance, DES‐G maintains a stable charge capacity of 365 mAh g^−1^ after 500 cycles at 0.5C with a capacity retention rate of 95.5%. In contrast, the capacity of C‐G after 500 cycles retains only 304 mAh g^−1^, and the capacity retention rate is 86.4%. More seriously, the capacity of W‐G after 300 cycles retains only 80 mAh g^−1^, which is almost completely scrapped at this stage. For the rate performance in Figure 4c, the DES‐G presents the specific charge capacities of 390.3, 363.1, 323.6, 231.8, and 96.3 mAh g^−1^ at the current densities of 0.1C, 0.5C, 1C, 2C, and 5C, respectively, which are all higher than those of C‐G and W‐G. It fully proves that DES‐G after regeneration not only has outstanding capacity and cycle performance, but also excellent rate performance, which is more suitable for use in high‐power electronic devices.

In the CV curves of Figure 4d and Figure S7 (Supporting Information), the DES‐G and C‐G both display a simple and obvious peak owing to SEI film formation at 0.7 V, but W‐G has a wide SEI film forming peak during 0.5–0.8 V. This wide peak indicates that impurities in graphite are involved in film forming reaction, and the film forming mechanism is more complex, which can be also confirmed by the large slope on the discharge curve. The oxidation and reduction peak of DES‐G in the 2–5 cycles are much coincident, which proves that the degree of reversibility is very high. In contrast, the CV curve of W‐G is not coincident, indicating that W‐G has serious polarization. In addition, the area enclosed by this CV curve is related to the specific capacity of the battery. In our work, the curve area of DES‐G is larger than that of W‐G, indicating that the specific capacity of DES‐G is higher than that of W‐G. In addition, the electrochemical impedance of DES‐G in Figure 3e is significantly lower than that of C‐G and W‐G.^[^ ^23^ ^]^ Generally, the lithium storage behavior of graphite can be divided into four electrochemical behaviors at different voltages: i) Lithium storage by adsorption at 0.16 V above. ii) Intercalation stage of LiC_24_→LiC_18_ from 0.1 to 0.16 V. iii, iv) Intercalation stage of LiC_18_→LiC_12_ and LiC_12_→LiC_6_, respectively.^[^ ^24^ ^]^ The characteristic peaks on d*_Q_*/d*_V_* often reflect the phase transition or structural transformation of graphite under a specific voltage. The Δ*V* of charge and discharge peak is related to the polarization of graphite during charge and discharge. In Figure 4f, the Δ*V* of DES‐G is 63 mV, compared with the Δ*V* of C‐G (84 mV), indicating that the electrochemical polarization of DES‐G is low and the reversibility is high. The d*_Q_*/d*_V_* of DES‐G and C‐G during 200 cycles are described respectively in Figure 4g and Figure S8 (Supporting Information). It is well known that its capacity during lithium storage is mainly related to stage (ii) and stage (iii) storage behavior. The integrated area of stage (ii) and (iii) determines the lithium storage capacity of graphite. Therefore, comparing the integral area of each cycle, it can be found that the cycle stability is closely related to deep storage. In addition, as the cycle continues, the peak of the discharge moves toward a lower voltage, indicating that more negative voltage potential is needed. More importantly, the reduction of the integral area indicates a decline in the lithium storage capacity, which leads to a corresponding reduction in the graphite cycle stability. In the meantime, compared with the regeneration methods of other teams in Figure 4h, as a clean and efficient recycling solvent, DES not only enables a short regeneration process, which is suitable for industrialization, but also enhances the capacity and cycle performance for the regenerated materials.^[^ ^25^ ^]^

**Figure 5:**
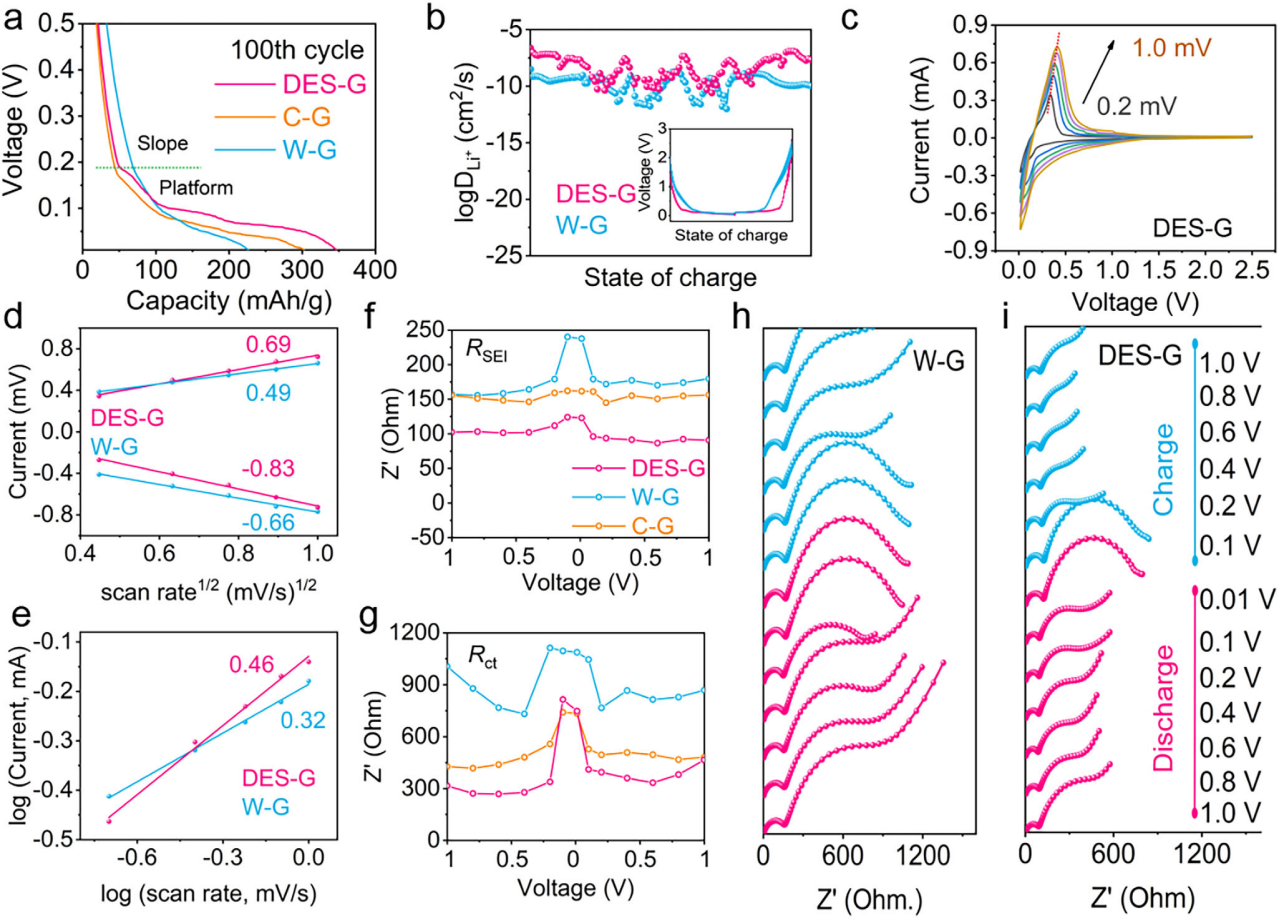
Kinetic analysis of graphite. a) Discharge curve at the 100th cycle of C‐G, W‐G and DES‐G. b) GITT tests of DES‐G and W‐G. c) CV curves of DES‐G at different sweep speeds of 0.2, 0.4, 0.6, 0.8, 1.0 mV/s. d) Linear relationship between *V* ^1/2^ and *I*. e) The logarithmic relationship between the peak current and scan rates. The f) *R*_SEI_ and g) *R*_ct_ of DES‐G, W‐G, and C‐G at different voltage. The in situ EIS profiles of h) W‐G and i) DES‐G.

To investigate the reasons for the increase in the capacity of DES‐G, the lithium storage mechanism of 100th cycle is analyzed, as shown in **Figure** **5**a. During the discharge process, the discharge capacity can be classified as slope capacity and platform capacity. Slope capacity is mainly caused by the adsorption of lithium by graphite edges and defects, and platform capacity is mainly attributed to the contribution of Li^+^ lithiated in the graphite layer. It is very noteworthy that the slope capacity of W‐G (before the cut‐off voltage of 0.16 V) is 71 mAh g^−1^, slightly higher than C‐G (46 mAh g^−1^) and DES‐G (56 mAh g^−1^), which is mainly due to the large specific surface area caused by holes and cracks on the surface of waste graphite. For the platform capacity (stage ii, iii and iv), the capacity of W‐G is only 153 mAh g^−1^, which is due to the existence of many impurities in the waste graphite layer, occupying the active site of graphite, so that its capacity is reduced. It is worth noting that DES‐G has a platform capacity up to 288 mAh g^−1^, even higher than the 253 mAh g^−1^ of C‐G. In the regeneration process of DES‐G, the impurities between graphite layers can be efficiently removed, and P is doped into the structure of graphite. The adsorption process of graphite edge contributes capacity to DES‐G, and furthermore P‐doping provides more active sites for lithiation.

To deeply explore the reason of the excellent electrochemical performance of DES‐G, GITT tests were performed to analyze the ion transport efficiency of graphite, as presented in Figure 5b and Figure S9 (Supporting Information). The Li^+^ transport efficiencies of DES‐G and W‐G appear a “W” type in GITT curves during charge and discharge, which belongs to the typical properties of graphite. The ion transport efficiency of DES‐G is between 10^−10^ and 10^−7^ cm^2^ s^−1^, much higher than that of W‐G (10^−12^−10^−8^ cm^2^ s^−1^), verifying that the obstruction in the Li^+^ transport path is much decreased. Figure 5c, and Figure S10 (Supporting Information) show CV curves of W‐G, C‐G, and DES‐G at different sweep speeds of 0.2, 0.4, 0.6, 0.8, and 1.0 mV s^−1^. With the increase in scanning rate, the redox peaks shift to higher voltage, and the integral area of the curve increases, which is a common phenomenon for all graphites. Figure 5d displays the functional relationship between *I* and *v* ^1/2^. It can be concluded that the functional slope of the oxidation peak is 0.69 and the reduction peak is −0.83 for DES‐G, which are higher than those of W‐G. It proves that the lithium diffusion kinetics of DES‐G are better than those of W‐G, and the conductivity is better. The kinetics of lithiated and de‐lithiated in graphite can also be calculated by Equation (1).

(1) $$\log\left(i\right)=\mathrm{blog}\left(v\right)+\log\left(a\right)$$

where *i* is the peak current and *v* is the scanning rate of CV curves. The slope of log(*v*)‐log(*i*) function can determine the lithium storage behavior of graphite. In Figure 5e, the slope of oxidation peak of DES‐G (0.46) is greater than the slope of W‐G (0.32), which is much closer to 0.5, reflecting the diffusion storage behavior. In DES‐G, Li^+^ can effectively diffuse inside the material and will not be deposited on the graphite surface. These results reveal that P‐doped and regenerated graphite can improve the kinetics of lithium storage, increase the diffusion rate of Li^+^ on graphite surface, realize the repair of waste graphite and improve its electrochemical performance.

Additionally, CV curves at different sweep speeds illustrate the lithium storage behavior of DES‐G during the cycles. The capacity of graphite can be divided into pseudocapacitance capacity and diffusion capacity. The contribution rate of pseudocapacitance of different graphites can be calculated according to the Equation (2).

(2) $$I=k_{1}v+k_{2}v^{1/2}$$

where *I* is the current, k_1_ and k_2_ are the proportional coefficients of pseudocapacitance and diffusion contribution respectively, and *v* is the scanning rate of CV curves. In Figure S11 (Supporting Information), through the calculation, the pseudocapacitance capacity is all greater than the diffusion capacity, and the pseudocapacitance behavior of DES‐G is all greater than that of W‐G at different sweep speeds.

Figure 5f–i and Figure S12 (Supporting Information) display the in‐situ EIS profiles of W‐G, C‐G and DES‐G at different voltages. In the range of 0.01–1 V, the impedance can be classified into two main components the SEI impedance (*R*_SEI_, the first semicircle) and charge transfer impedance (*R*_ct_, the second semicircle).^[^ ^26^ ^]^ It can be found that the *R*_SEI_ of W‐G is significantly greater than that of W‐G, indicating that the SEI film formed by DES‐G can transfer Li^+^ more quickly. Meanwhile, the *R*ct is generally related to the state of charge (SOC) and increases with the depth of discharge. In W‐G, the *R*ct always presents a large value, and the continuous high impedance may greatly hinder the ion migration dynamics, thereby reducing the performance of battery. By comparison, the *R*ct of DES‐G changes with SOC, and only increases suddenly at the low voltage of 0.1 V with high SOC, and afterward recovers with the charging process, confirming again that the overall reversibility of DES‐G is excellent. In addition, the changes of DES‐G appear the similar trend and range as those of C‐G.

### 2.4 Mechanism Analysis

**Figure 6:**
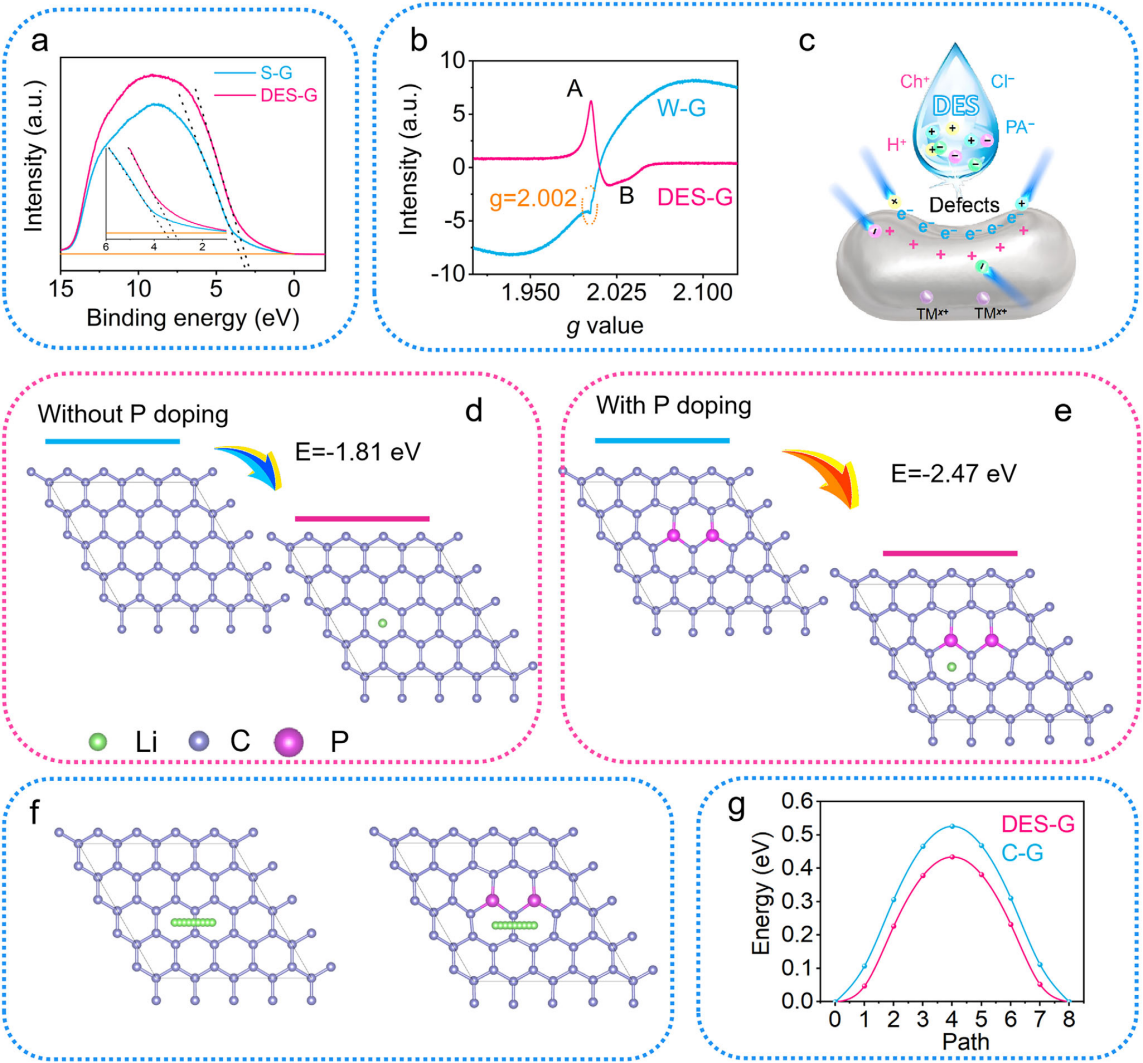
Mechanism analysis of DES‐G. a) The UPS spectra of W‐G and DES‐G. b) The EPR profiles of W‐G and DES‐G. c) Schematic diagram of charged ions in DES attacking graphite defects. The calculated adsorption energy of d) traditional C‐G and e) DES‐G by DFT. f) The Li^+^ transport path of C‐G and DES‐G. g) The calculated migration energy barrier of Li^+^ in C‐G and DES‐G by DFT.

The valence band of graphite is analyzed using ultraviolet photoelectron spectroscopy (UPS), which is related to the conductivity. In **Figure** **6**a, the DES‐G has a narrower band gap and a valence band closer to Fermi level after regeneration, which is conducive to the rapid exchange of electrons between the band gaps.^[^ ^27^ ^]^ Therefore, it can also be proved that P‐doped graphite after regeneration using DES has higher electrical conductivity and is more beneficial for electron exchange. In Figure S13 (Supporting Information), the specific surface area of DES‐G after regeneration is 5.7 m^2^ g^−1^, which is higher than that of C‐G and W‐G. Appropriate surface area and pore volume can provide more active sites, which is conducive to ion transport and electrolyte penetration, and finally achieve the effect of improving the storage capacity of Li^+^.

Additionally, as displayed in Figure 6b, the unpaired electron spin is probed in graphite using electron paramagnetic resonance (EPR). Generally, the EPR profile of graphite appears as a pair of asymmetric peaks, which is derived from the fast movement of the electrons and the skin effect. The peak up is defined as graphite A, the peak down as graphite B, and the intensity value of A/B is related to the conductivity of graphite.^[^ ^28^ ^]^ However, it is obvious that the baseline of W‐G is unstable, and the graphite A and B peaks are buried, which is caused by the magnetic element contained in W‐G. In contrast, the DES‐G baseline is stable, proving that DES‐G does not contain magnetic elements, and the peak shape presents the characteristics of a complete graphite peak. The A/B values of DES‐G is 2.18, confirming that the regenerated materials have better conductivity. Another major discovery in the inclined baseline of W‐G is that a defect peak emerges at g = 2.002, verifying that the defects occur on the W‐G. A model of charged DES attacking charged defects is proposed in Figure 6c, in which electrostatic interaction is the main driving force for P doping and metal ion removal. In the process of charging and discharging, Li^+^ ions are constantly embedded and removed between the graphite layers, thereby causing a carbon atom missing. Correspondingly, an electron is leaved that can participate in the π bond and stabilize the graphite structure, forming a vacancy structure. Therefore, the interior of waste graphite is embedded with positively charged transition metal cations, and on the surface are positively charged vacancy and negatively charged vacancy electrons.^[^ ^29^ ^]^ As mentioned above, DES is rich in tanglesome charged anions and cations, such as Cl^−^, Ch^+^, H^+^ and PA^−^, etc. During the attack‐to‐graphite process, Cl^−^ anions chelate with charged metal ions for the purpose of impurity removal, and meanwhile the cations bind to the electrons on the vacancy surface to neutralize the electrons. The negatively charged P‐rich group attacks the positively charged vacancy to achieve the purpose of P‐doping. In contrast, C‐G has no defects on its surface so it cannot be attacked by DES. As demonstrated in Figure S14 (Supporting Information), C‐G‐DES (C‐G treated via DES) has no significant functional groups such as C─P and P═O, confirming that there is no active site attacked by charged particles.

Generally, the lithium storage process of graphite can be divided into two steps: the Li^+^ in the electrolyte is first adsorbed by the graphite surface, and then the Li^+^ migrates into the graphite interior. The adsorption of Li^+^ by graphite will affect the interface reaction on the electrode surface, and the migration energy barrier of lithium in graphite directly determines the ion transport efficiency. Therefore, the adsorption energy and migration energy barrier are the key indicators in the electrode reaction kinetics, which affect the energy density, cycle stability, and safety of the battery. The changes of adsorption properties and migration energy barrier of graphite regenerated by DES are investigated by theoretical calculation.^[^ ^30^ ^]^ From Figure 6d,e, the adsorption energy of traditional C‐G for lithium is −1.81 eV, while in contrast, the adsorption energy of graphite regenerated using DES is reduced to −2.47 eV due to the presence of phosphorus doping. Such a low adsorption energy indicates that DES‐G can better adsorb Li^+^ and accelerate the kinetics of surface adsorption. Meanwhile, the migration energy barrier of Li^+^ in C‐G and DES‐G is compared, as presented in Figure 6f,g. The diffusion paths of the two models have similar characteristics, that is, the energy of the initial state and the final state is low, and the diffusion potential of the middle state is very high. In addition, the diffusion energy barrier of DES‐G is 0.43 eV, lower than that of C‐G (0.52 eV), revealing that the diffusion resistance of Li^+^ in the regenerated graphite is reduced. The low diffusion barrier can enhance ion dynamics, resulting in better rate properties. After DES treatment, the graphite is doped with phosphorus, and phosphorus has a larger atomic radius, so that the regenerated graphite has a larger interlayer distance, promoting the accumulation of charge and enhancing the diffusion kinetics and efficiency.

**Figure 7:**
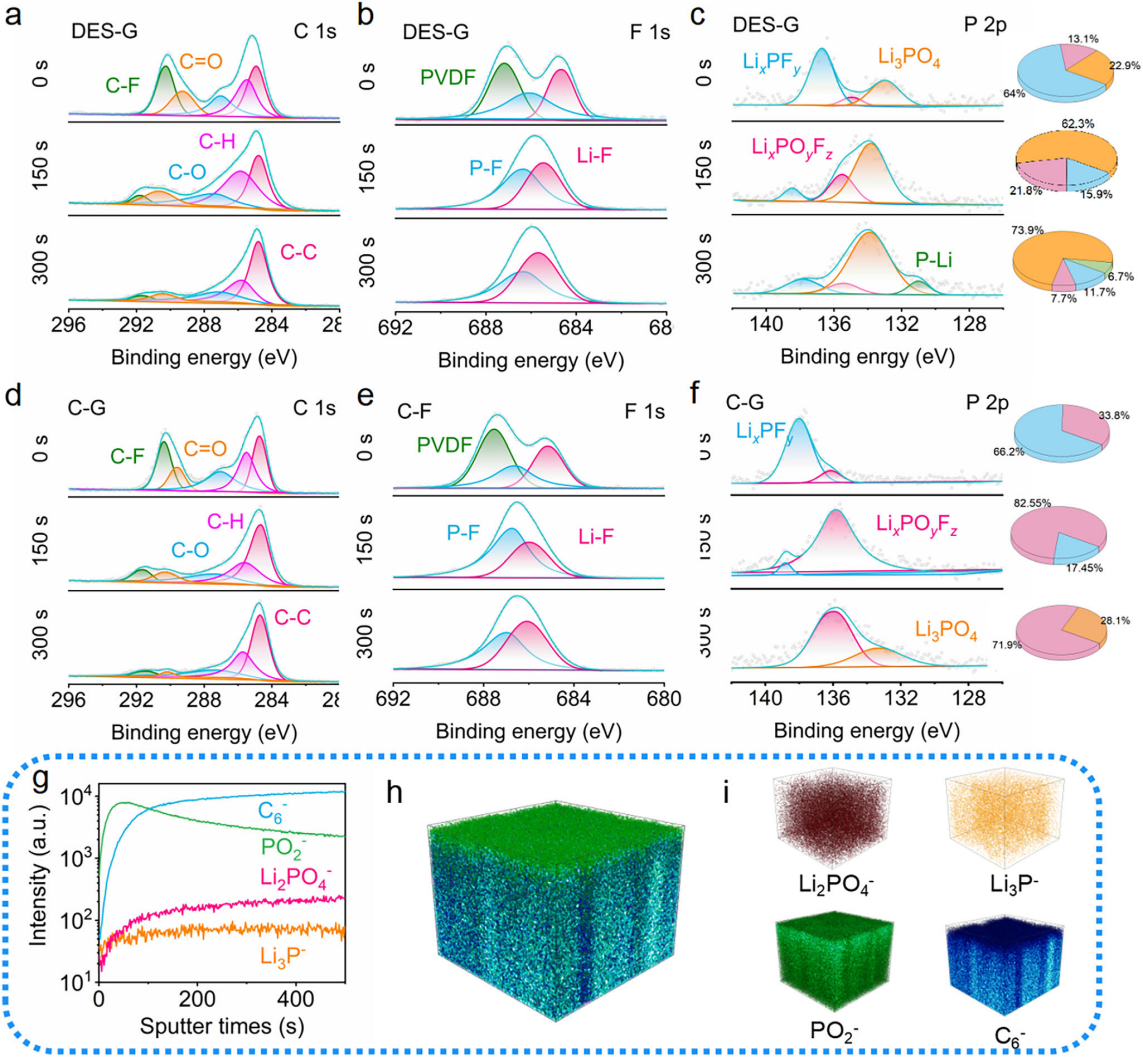
SEI analysis of DES‐G and C‐G. Depth sputtered XPS spectra of SEI film of a) C 1s, b) F 1s, and c) P 2p in DES‐G. Depth sputtered XPS spectra of SEI film of d) C 1s, e) F 1s, and f) P 2p in C‐G, the illustration shows the content of P in different states (blue: Li*_x_* PF*_y_*, pink: Li*_x_* PO*_y_* F*_z_*, orange: Li_3_PO_4,_ and green: Li_3_P). g) Negative‐mode TOF‐SIMS depth profiles of Li_3_PO_4_^−^, Li_3_P^−^, C_6_^−^, and PO_2_^‐^ in DES‐G and their corresponding 3D renderings, h) mixture, i) brown: Li_3_PO_4_^−^, yellow: Li_3_P^−^, green: PO_2_^‐,^ and blue C_6_^−^.

The solid electrolyte interphase film (SEI) formed between the electrode and electrolyte in the early cycle can stabilize the anode, inhibit the decomposition of electrolyte, selectively screen Li^+^, and promote the transmission of Li^+^, which composition and physicochemical properties affect the electrochemical performance of the battery.^[^ ^31^ ^]^ **Figure** **7**a–f and Figures S15–S18 (Supporting Information) display the depth‐sputtered XPS spectra of SEI film of DES‐G, C‐G, and W‐G after cycling. In C1s spectra, the characteristic peaks belonging to C─F, C═O, C─O, C─H, C─C can all be observed. PVDF is the binder, which mainly exists on the electrode surface of DES‐G, C‐G, and W‐G. C═O, C─O, C─H are originated from the decomposition products (lithium alkoxy and lithium carbonate) of electrolyte, which exist on the electrode surface of DES‐G, C‐G and W‐G and the outer surface of SEI film, while the contents of lithium alkoxy and lithium carbonate dominated by C═O and C─O at the inner interface of SEI film is extremely low. There is no obvious difference between DES‐G, C‐G, and W‐G, which all follow the law of SEI film formation. A similar phenomenon also displays in F1s spectra, and the electrode surface of DES‐G, C‐G, and W‐G contains PVDF binder and the SEI film composed of Li*_x_* PF*_y_*, Li*_x_* PO*_y_* F*_z,_* and LiF. LiF is the main component of SEI film with higher ionic conductivity, lower diffusion energy, and higher surface energy. The content of LiF in the SEI film of DES‐G is higher than that of the decomposition product F‐P, but there is no obvious difference in the SEI film of C‐G. In P2p spectra. An obvious difference can be observed in the SEI film of DES‐G, C‐G, and W‐G. First, Li*_x_* PF*_y_*, Li*_x_* PO*_y_* F*_z,_* and Li_3_PO_4_ emerge on the surface of the SEI film of DES‐G. In the SEI film of DES‐G, the material surface is mainly composed of Li*_x_* PF*_y_*, while Li_3_PO_4_ only accounts for 22.9%. As the etching progresses, the composition of the SEI film is mainly Li_3_PO_4_, and the contents of Li_3_PO_4_ are 62.3% and 73.9% respectively, at 150 and 300 s. Moreover, at a very deep position, a content of 6.7% Li_3_P has been newly discovered. Among them, Li_3_PO_4_ has a lower working function of only 5.2 eV, which is lower than that of Li_2_CO_3_ (6.0 eV) and LiF (7.6 eV). It can easily provide electrons and act as electron donors to adsorb solvated Li^+^, thereby reducing the interaction between Li^+^ and solvent molecules, which is conducive to help Li^+^ to desolvate from its solvated sheath.^[^ ^3b^ ^]^ Second, Li_3_P can be found in the inner layer of SEI film of DES‐G, which is a fast ionic conductor. It can adsorb solvent molecules, weaken the interaction between Li^+^ and solvent, promote Li^+^ desolvation, to avoid solvent co‐insertion of graphite.^[^ ^32^ ^]^ By comparison, for the SEI film of C‐G, trace Li_3_PO_4_ is only present in the inorganic layer, and Li_3_P does not exist. Detailed information on the spectra of O 1s and Li 1s is provided in the supporting information, also confirming that the SEI film of DES‐G is inorganics inside and organics outside.

Figure 7g–m and Figure S19 (Supporting Information) present the Time‐of‐Flight Secondary Ion Mass Spectrometry (TOF‐SIMS) of DES‐G after cycling. The etching area is 100 × 100 µm^2^, and the etching time is 500 s. In the mass spectrum, the electrolyte decomposition products such as PO_2_^−^ and PF^−^ can be found on the surface of DES‐G. With the extended etching time, many inorganic groups such as Li_3_PO_4_^−^, Li_3_P^−^, LiF^−,^ and Li_2_O^−^ appear, further confirming the existence of Li_3_PO_4_ and Li_3_P in the SEI film. Li_3_PO_4_ and Li_3_P can accelerate charge transport dynamics and resist the co‐intercalation of low melting point solvent molecules. In addition, Li_3_P acts as a fast ionic conductor assisting Li^+^ to pass through the SEI, and eventually promoting the high capacity and high stability of DES‐G.

### 2.5 Economic and Environmental Analysis

Currently, the mainstream industrial method for the recovery of waste graphite can be divided into two steps: first, using strong acids to leach metal ions embedded in the graphite, and then using high‐temperature graphitization to repair its structure. This method will produce a large amount of wastewater and acid in the process of acid leaching. In the graphitization process, the graphitization temperature is 2500–3000 °C, which will produce plentiful energy consumption. In short, this process will cause a huge threat to the environment, and the huge energy consumption is not in line with the concept of green development. In recent years, researchers have attached great significance to oxidizing and peeling off waste graphite to produce higher‐value graphene or graphene oxide owing to the characteristics of waste graphite with larger layer spacing. The product obtained by this process possesses high economic value, but the market is small, and the graphene market cannot digest the huge amount of waste graphite. In addition, in the process of graphene preparation, many strong acids and strong oxidants are needed, and Mn_2_O_7_ and other dangerous by‐products may be produced, which is not conducive to industrial production. From the perspective of carbon footprint, the first method has a large amount of carbon emissions in the calcination process, while the second process uses huge raw materials and has a large amount of carbon emissions at the material end.

**Figure 8:**
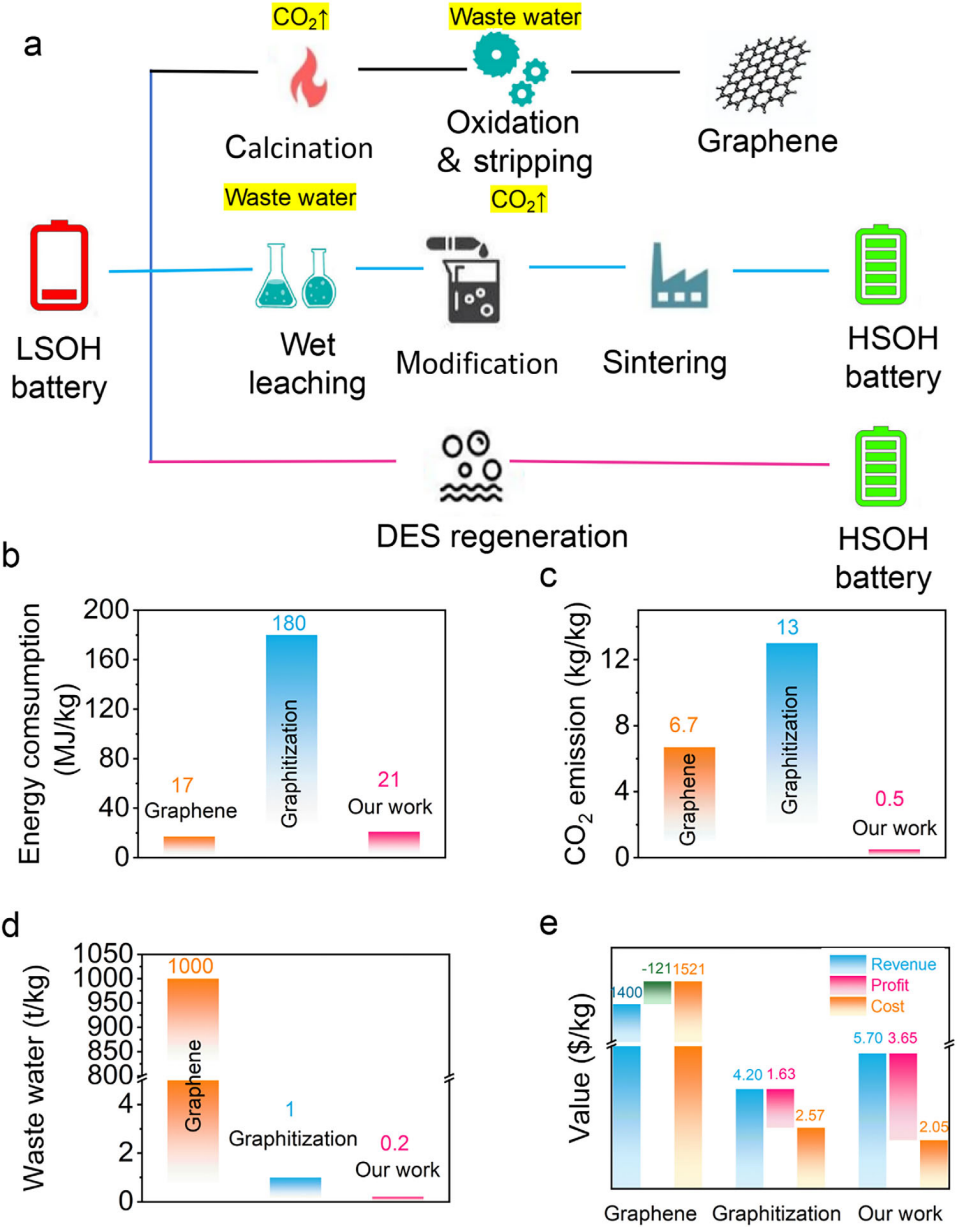
Economic and environmental analysis of three models. a) Process Flow Diagrams for three methods. b) Energy consumption, c) Carbon emission, d) Wastewater, e) Cost and profit analysis of three models.

To evaluate the environmental and economic benefits of these methods, three models are constructed, namely graphene preparation, high temperature graphitization, and DES‐regeneration in our work, as displayed in **Figure** **8**.^[^ ^33^ ^]^ In the graphene preparation, the binder and conductive carbon are first calcined to remove, then the waste graphite is oxidized by concentrated sulfuric acid, phosphoric acid, and potassium permanganate etc., and finally the graphene is obtained by stripping in an ultrasonic process. In the high temperature graphitization, the metal ions are first leached out with inorganic acids such as sulfuric acid or hydrochloric acid etc., and the leached products are put into the graphitization furnace, and the graphite is graphitized at a temperature of 2500–3000 °C. In the DES‐regeneration, DES composed of phytic acid and choline chloride is applied for one‐step leaching and doping, and the waste graphite is directly regenerated into high‐performance P‐doped graphite. The types and quantities of raw materials and technological conditions used in these three methods are presented in Tables S1–S3 (Supporting Information), respectively. The controlled temperature of graphene in the oxidation process is 0–5 °C, the temperature required for graphitization is 2500–3000 °C, and the temperature required for DES‐regeneration is 80 °C. The energy consumption of graphitization process is up to 180 MJ kg^−1^, which is much higher than the energy consumption of DES‐regeneration in our work. Therefore, combined with the carbon emission during the preparation of raw materials, our process displays the lowest carbon emission, only 0.5 kg/kg graphite. In addition, our process produces the lowest amount of wastewater.

To prove that our process is more conducive to industrial production, three methods are analyzed economically. Although the economic value of the preparation of graphene is higher, the input of process equipment and raw materials is larger, and the profit generated by the final accounting is negative. Combined with the market demand for graphene, the conclusion is that the process of preparing graphene is not suitable for commercialization. The profit of our process is 3.65 $ kg^−1^, which is higher than the graphitization process. In addition, our process also has the advantages of short process flow, low energy consumption, green environmental protection, etc.

## 3 Conclusion

In sum, direct upcycling of waste graphite and in‐situ phosphorus doping are realized under mild conditions by use of DES, and a mechanism of in‐situ doping by charged ions attacking vacancies is disclosed. The PDF calculation shows that the bond length of regenerated graphite increases, confirming that phosphorus is introduced into the graphite lattice. Due to phosphorus doping, regenerated graphite possesses the characteristics of high capacity of 365 mAh g^−1^ after 500 cycles at 0.5C, high conductivity, high efficiency of lithium‐ion transport, fast dynamics, and good cycle stability. In addition, phosphorus doped in graphite can participate in the reconstruction process of SEI film, forming phosphate‐rich SEI that accelerates lithium ion desolvation and avoids co‐embedding of solvent molecules. More importantly, the regeneration process has high economic benefits and low environmental impact, can be quickly promoted to industrialization, and can realize the regeneration of waste graphite with high efficiency, high economic benefits, and low environmental pollution.

## Conflict of Interest

The authors declare no conflict of interest.

## Author Contributions

X.L. and S.L. contributed to this work equally. The manuscript was written through contributions of all authors. X.L. and J.L. designed the experiments. X.L.; S.L. and R.Z. carried out the chemical lithiation, and electrochemical tests. J.P. and J.W. analyzed the experimental results of X‐ray diffraction/spectroscopy and Raman spectroscopy, respectively. J.P. calculated the economic and environmental impacts of the recycling process using EverBatt. The original idea was conceived by X.L. and J.M., who supervised all the experiments. W.L. and J.L. discussed the results and revised the manuscript. All authors discussed the results and gave approval to the final version of the manuscript.

## Supporting information



Supporting Information

## Data Availability

The data that support the findings of this study are available from the corresponding author upon reasonable request.
