# Supplementary material for: Sustainable and Direct Upcycling of Waste Graphite Anodes via Deep Eutectic Solvents
Source: Adv Sci (Weinh). 2025 Aug 18;12(41):e06637. doi: 10.1002/advs.202506637 (PMC12591146; doi:10.1002/advs.202506637)
Supplement: Supplementary file 1 — Supporting Information [file ADVS-12-e06637-s001.docx]

**Supporting Information**

**Sustainable and Direct Upcycling of Waste Graphite Anodes via Deep Eutectic Solvents**

Xue Liu,^#,a,b^ Shaoqing Liu,^#,a^ Junhan Pu,^a^ Rong Zeng,^a^ Tao Wang,^a^ Jiashen Meng,^c^ Jean-Jacques Gaumet,^d^ Wen Luo,^*,b,c^ Jianwen Liu^*,a^

*^a^ College of New Energy and Electrical Engineering & College of Chemistry and Chemical Engineering & Ministry of Education Key Laboratory for Green Preparation and Application of Functional Materials, Hubei University, Wuhan 430062, P. R. China*

*^b^ School of Physics and Mechanics, Wuhan University of Technology, Wuhan 430070, P. R. China*

*^c^ State Key Laboratory of Advanced Technology for Materials Synthesis and Processing, School of Materials Science and Engineering, Wuhan University of Technology, Wuhan 430070, P. R. China*

*^d^ Laboratoire de Chimie et Physique: Approche Multi-échelles, des Milieux Complexes (LCP-A2MC), Institut Jean Barriol, Université de Lorraine, Metz, 57070, France*

^#^ Xue Liu and Shaoqing Liu contributed to this work equally.

* Corresponding authors. E-mail: luowen_1991@whut.edu.cn (Prof. & Dr. Wen Luo); jianwen@hubu.edu.cn (Prof. & Dr. Jianwen Liu)

**Experimental section**

**Experimental preparation and raw materials**

Spent LIBs were provided by Hubei WanRun New Energy Co., Ltd., and firstly soaked in 10 g/L NaCl solution for full discharge for 24 hours. Then they were disassembled, separated, and screened to obtain waste graphite powders after drying. The metal ion content in waste graphite was measured by inductively coupled plasma-optical emission spectrometry (ICP-OES). As listed in Table S4, the main metal (Li, Ni, Co, Mn, Fe, Al, Cu) and non-metal (F, P) impurities in waste graphite can be largely removed after DES regeneration. PA (70% aqueous solution) and ChCl (purity of 98%) were purchased from Aladdin Reagent, and other raw materials used in this experiment were all analytically pure.

**Regeneration of waste graphite by DES**

Before regeneration, acetylene black, binder and organic compounds on the surface of waste graphite were roasted by heating the powders at 600 °C for 5 hours in a muffle furnace. Firstly, 0.01 mol of ChCl and 0.01 mol of PA were mixed in a 30 mL glass bottle and heated in an oil bath at 80 °C until it is transparent to form DES. Subsequently, 0.5 g waste graphite was added to the above as-prepared DES and heated at 80 °C for 10 hours. After centrifugation, the solid was washed with deionized water for 3 times and ethanol for 3 times in turn, to obtain the regenerated graphite, which was labeled as DES-G.

**Structure characterizations**

The contents of metal ions in waste graphite were determined by inductively coupled plasma emission spectrometry (ICP-OES). The phase structure is characterized by X-ray diffractometry (XRD, D/MAX-IIIC, Bruker). Besides, surface morphologies and particle size distributions of waste and regenerated graphite were characterized by scanning electron microscopy (SEM, SIGLA-500, Germany) and field emission scanning electron microscopy (FESEM, JSM-7500FA, USA). For further analysis the lattice fringes and crystal face characteristics of waste and regenerated graphite, transmission electron microscopy (TEM) be used. Meanwhile, the content and distribution of elements were analyzed by X-ray energy dispersive spectroscopy (EDS). X-ray photoelectron spectroscopy (XPS, Escalab 250Xi, Thermo Fisher Scientific) was used to characterize the binding energies, valence states and valence components of waste and regenerated graphite. The compositions, structures, and relative contents of waste and regenerated graphite were analyzed by laser Raman spectrometer (RM2000, Renishaw). Thermogravimetry (TG, Mettler TGA2 and Fourier Transform Infrared Spectroscopy (FTIR, Thermerfield Is50) were combined to analyze the thermodynamic stability of DES, and nuclear magnetic resonance (NMR, Bruker Avance Neo400) was used to analyze the changes of functional groups in DES.

**Battery assembly and electrochemical testing**

When manufacturing batteries, the graphites including waste graphite (W-G), regenerated graphite (DES-G) and commercial graphite (C-G), 4 wt.% polyvinylidene fluoride (PVDF, dissolved in NMP) and acetylene black were mixed and coated on aluminum foil with a mass ratio of 8:1:1 and transferred to a vacuum oven at 120 °C overnight drying. The next day, the thin aluminum covered with material were cut into a round sheet with a diameter of 8 mm, which mass loading is 2.1−3.2 mg/cm^2^. The experimental batteries were assembled with the DES-G, C-G, W-G as the anodes, lithium plates as the anode, 1 M lithium hexafluorophosphate (LiPF_6_) dissolving into DMC:EC:EMC=1:1:1 vol.% as the electrolyte and Celgard 2400 membrane as the septum. Assembly of the battery system takes place in a glovebox filled with argon, which contains less than 0.01ppm of water and oxygen. The cycle and rate performance tests (1C=372 mAh/g) were carried out on the NEWARE Battery Testing System (CT-4008T-5V50Ma-164, Shenzhen, China) in the voltage of 2.5−0.01 V. GITT tests were carried out also on the NEWARE system with the charging rate of 0.1C, the pulse time of 10 minutes and the rest time of 40 minutes. Cyclic voltammetry (CV), were tested on Donghua Electrochemical workstation (DH7003, Jiangsu, China) and electrochemical impedance (EIS) were tested on the same electrochemical workstation with the scanning rate of 0.1 mV/s and the frequency atmosphere of 100 kHz−0.1 Hz, respectively.

**Theoretical calculations**

The Vienna ab initio simulation package (VASP) based on the DFT was used to perform all calculations. The projector-augmented wave (PAW) method was conducted to describe the interactions between ion cores and valence electrons. The Perdew-Burke-Ernzerhof functional was adopted for the calculation of exchange and correlation. The cutoff energy was set to 420 eV. All structures were optimized until the energy was converged to 1.0×10^−5^ eV and the force to 0.01 eV/Å. Moreover, the CI-NEB method was adopted for the diffusion paths of Li^+^.

**Statistical Analysis**

All the data in the article were original data. Even though the XPS data was subjected to peak fitting, the original data was still retained in the figure.


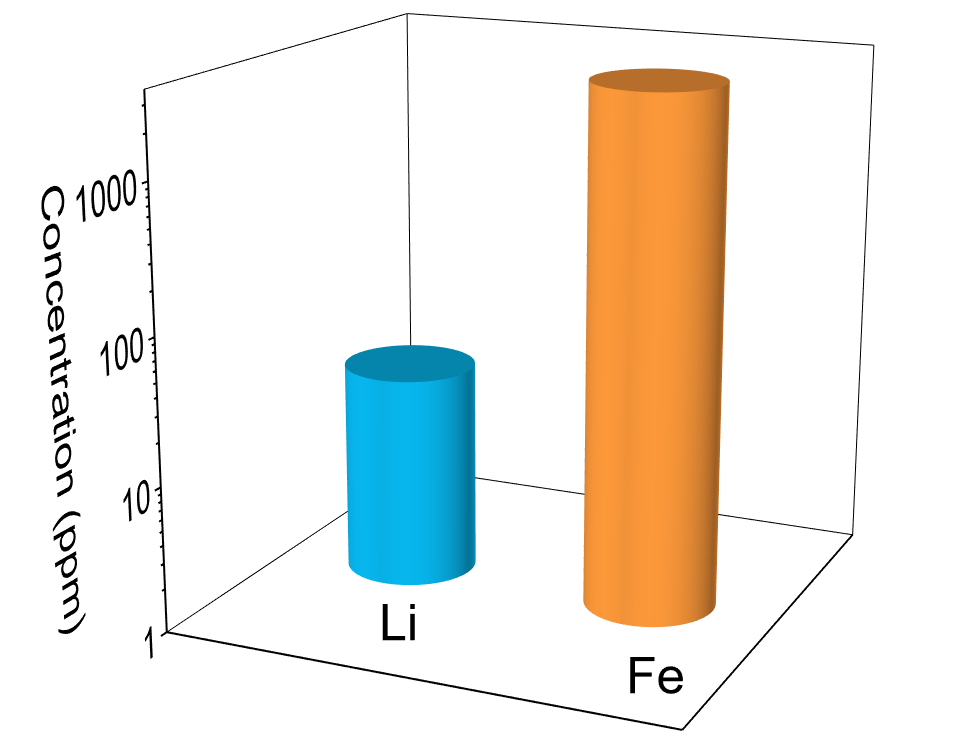


**Fig. S1.** The ICP date of the DES leaching solution.





**Fig. S2.** TG and DTG curves of the mixtures tested by TG-FTIR.





**Fig. S3.** The FTIR spectra of W-G and DES-G.


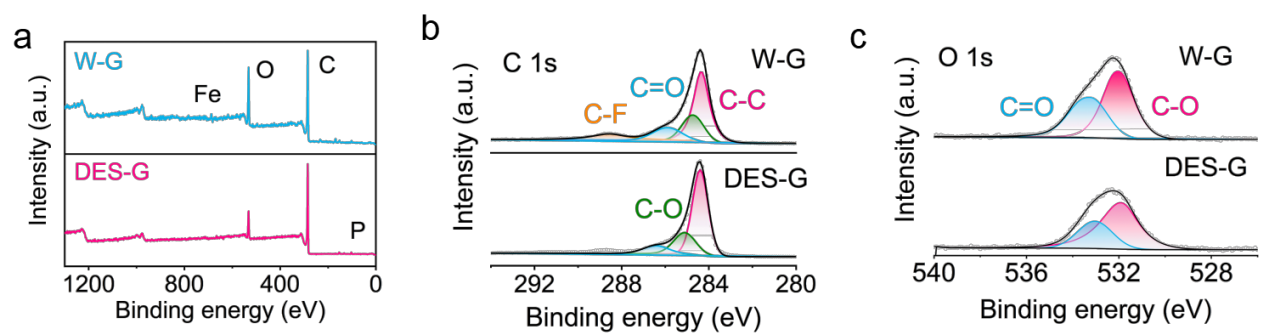


**Fig. S4.** (a)The XPS spectra of W-G and DES-G and (b) C1s, (c) O1s.


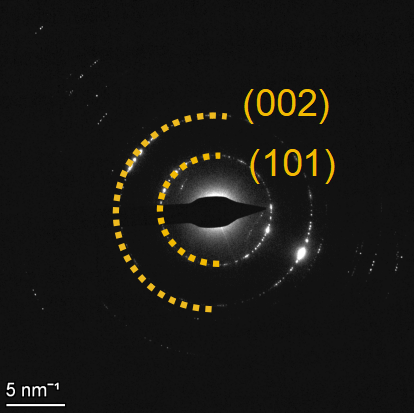


**Fig. S5.** Selected area electron diffraction (SAED) images of DES-G.


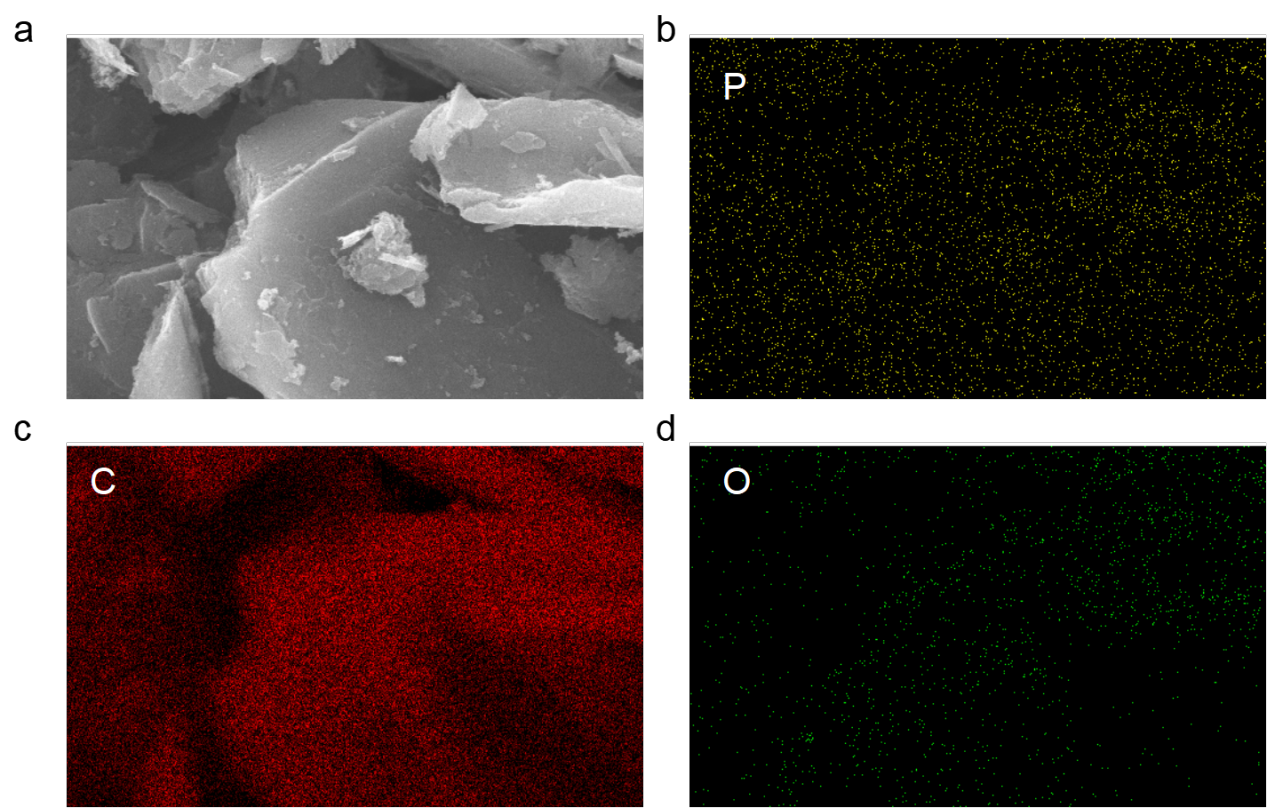


**Fig. S6.** SEM images of (a) DES-G after DES regeneration, and element distributions including C, O and P in DES-G (b−d) (Red: C, Yellow: P, Green: O).








**Fig. S7.** The CV curves of C-G and W-G at a scan rate of 0.1 mV/s.

1→200





**Fig. S8.** The dQ/dV of C-G during 200 cycles.


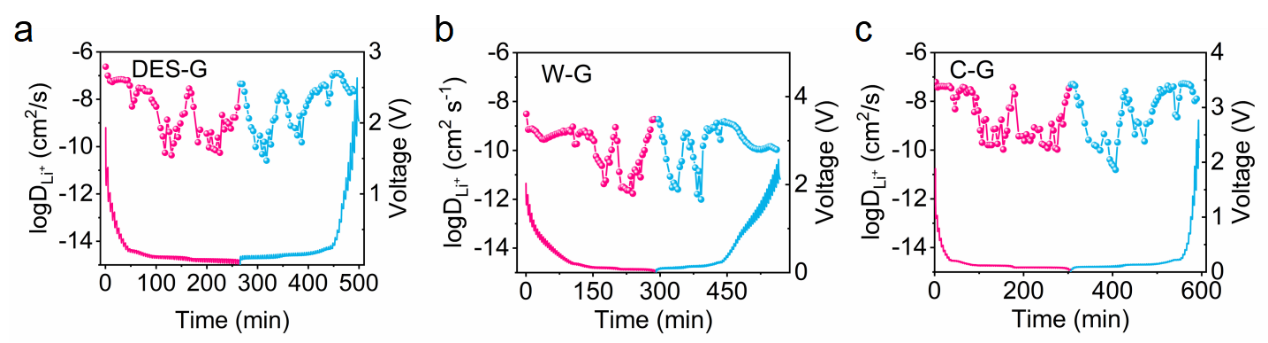


**Fig. S9.** GITT testings of (a) DES-G, (b) W-G and (c) C-G.


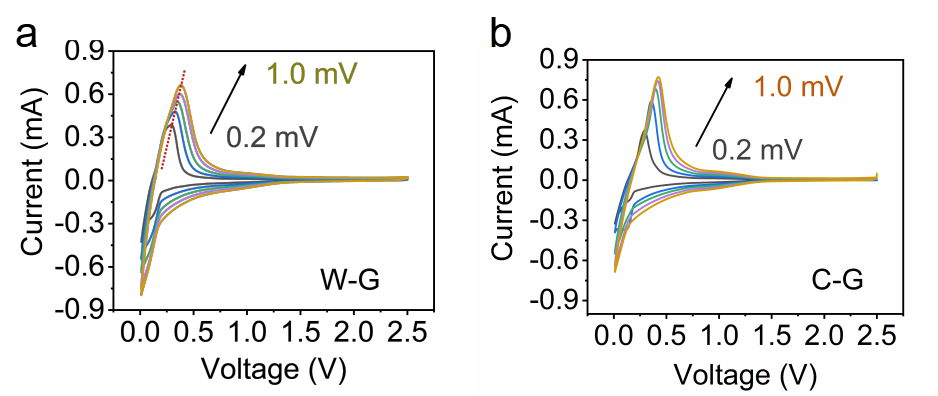


**Fig. S10.** The CV curves of (a) W-G and (b) C-G at different sweep speeds of 0.2, 0.4, 0.6, 0.8, 1.0 mV/s.


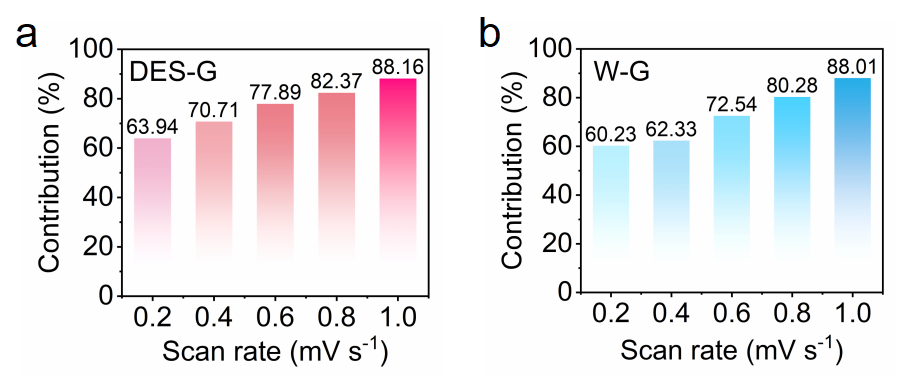


**Fig. S11.** Pseudocapacitance contribution of DES-G and W-G.





**Fig. S12.** In-situ EIS profiles of C-G.


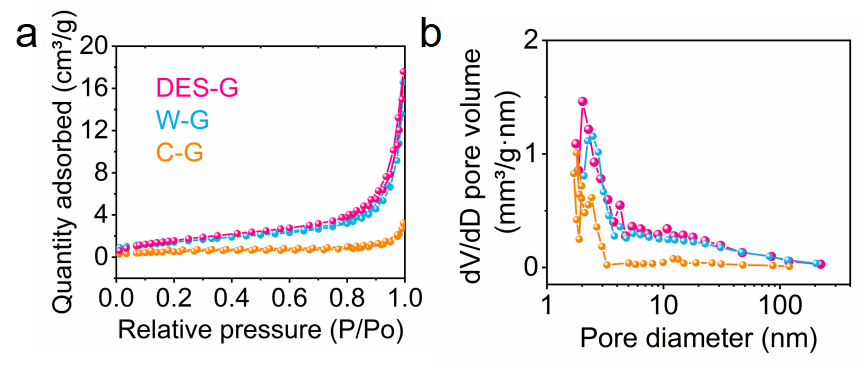


**Fig. S13.** (a) The specific surface areas and (b) pore diameters of DES-G, W-G and C-G.





**Fig. S14.** FTIR spectra of C-G-DES.


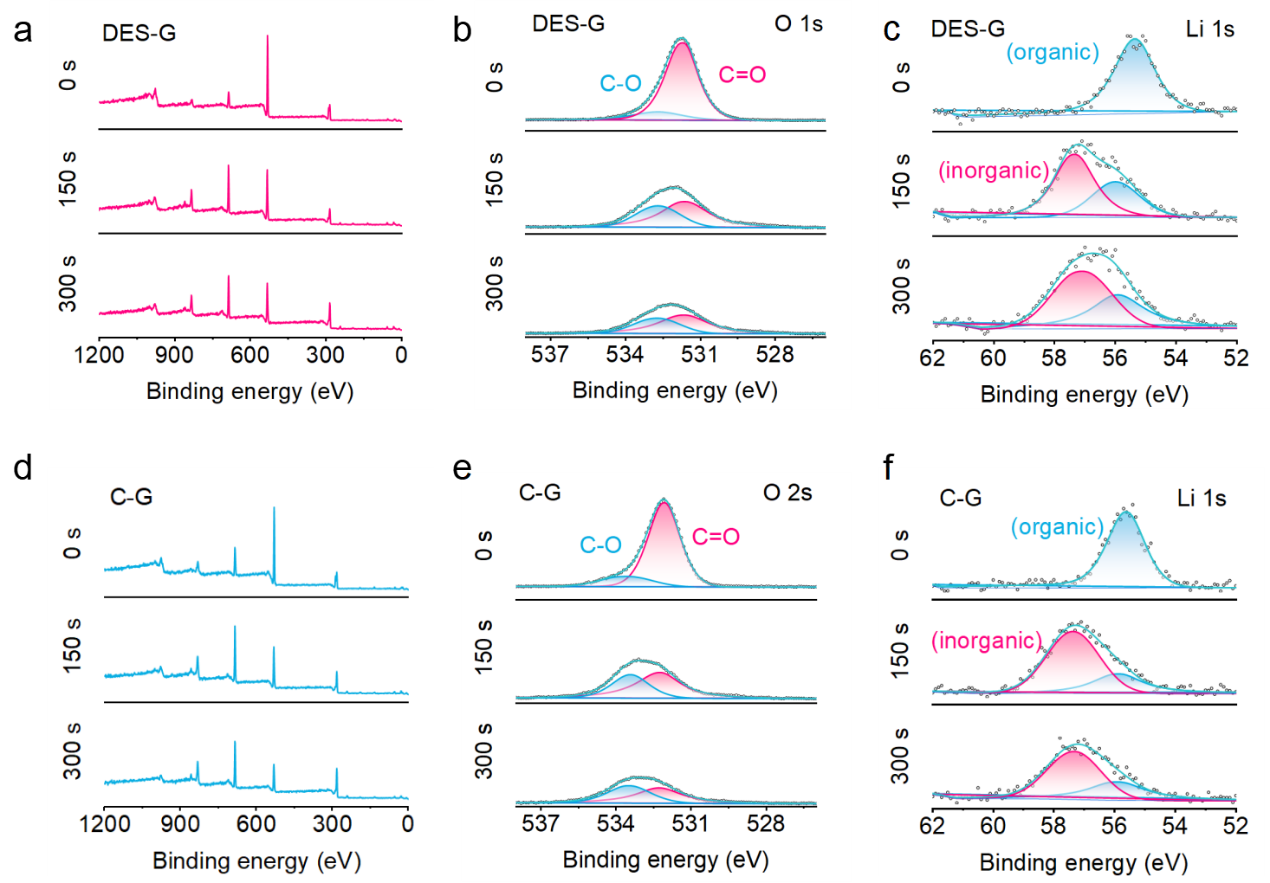


**Fig. S15.** Depth sputtered XPS spectra of SEI film: (a) full spectrum, (b) O 1s and (c) Li 1s in DES-G. Depth sputtered XPS spectra of SEI film: (d) full spectrum, (e) O 1s and (f) Li 1s in C-G.


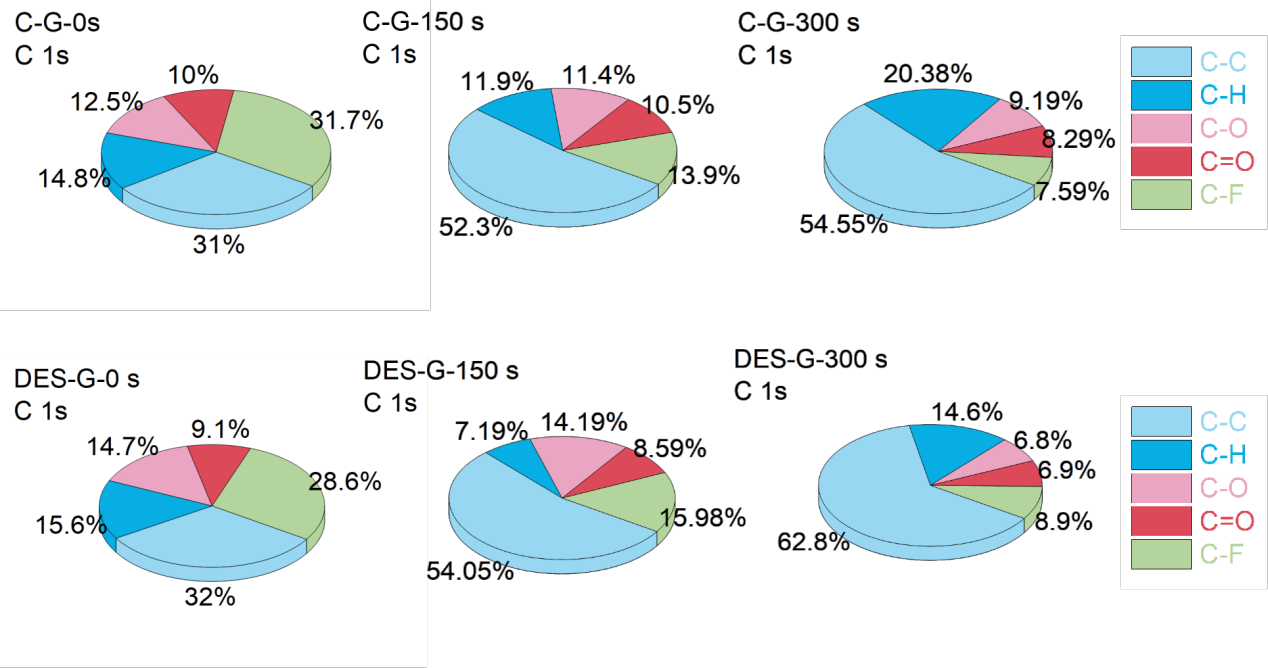


**Fig. S16.** The numerical form Depth sputtered C 1s XPS data of SEI film of C-G and DES-G.


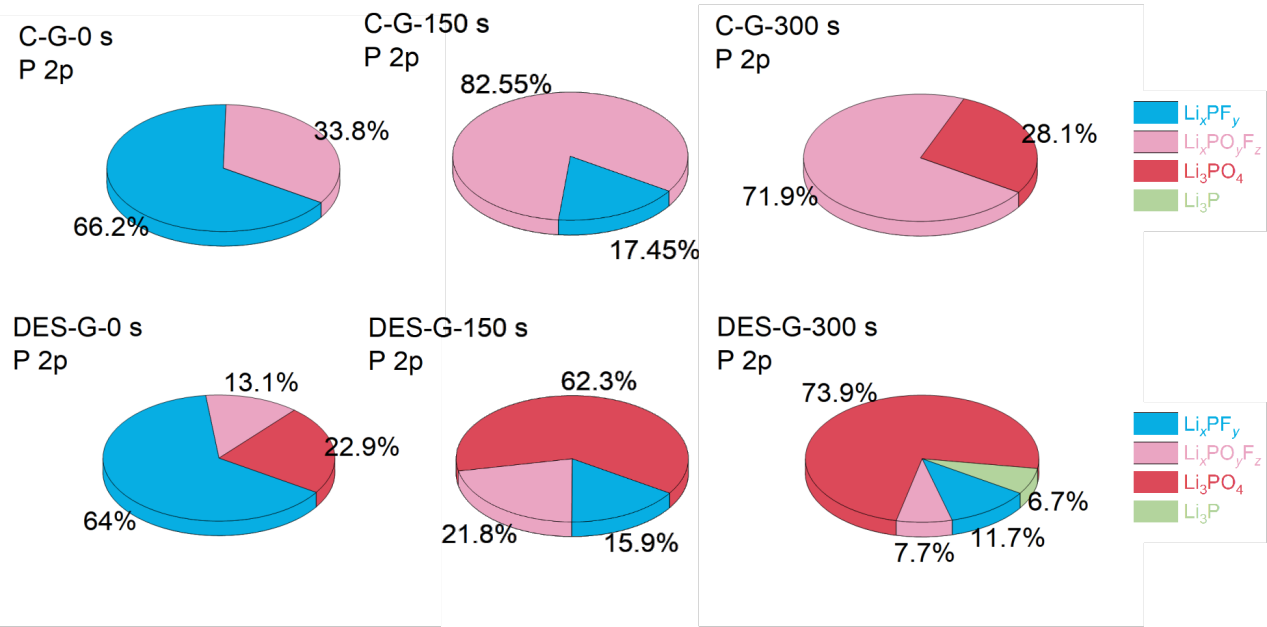


**Fig. S17.** The numerical form Depth sputtered P 2p XPS data of SEI film of C-G and DES-G.


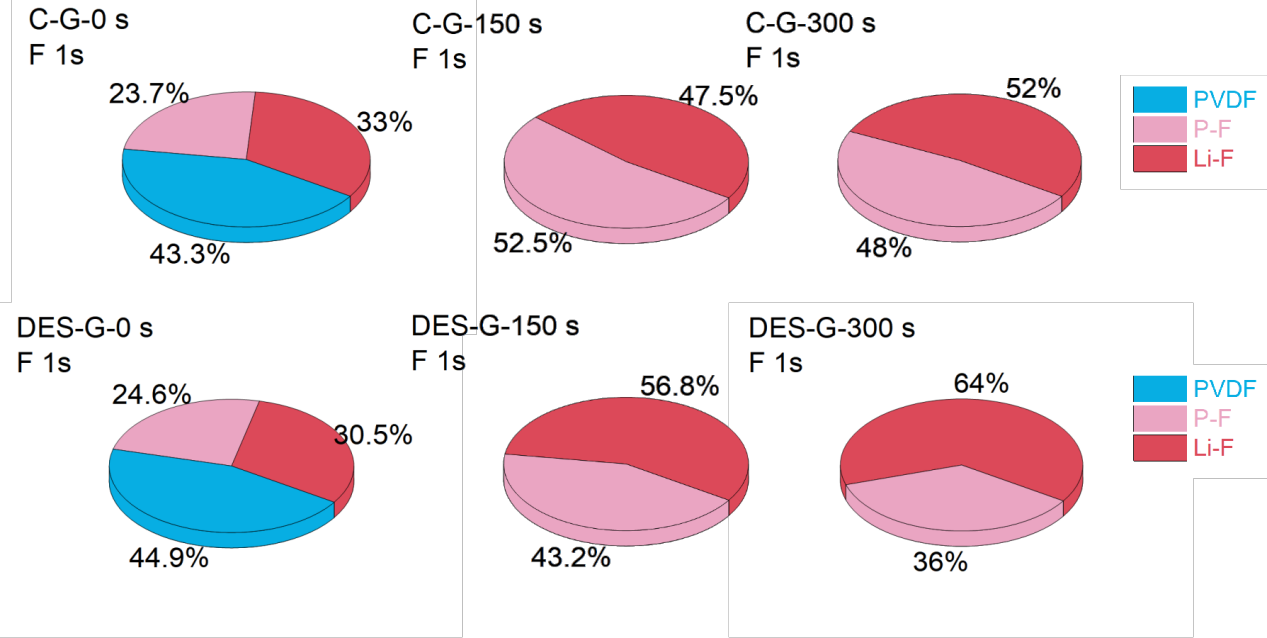


**Fig. S18.** The numerical form Depth sputtered F 1s XPS data of SEI film of C-G and DES-G.


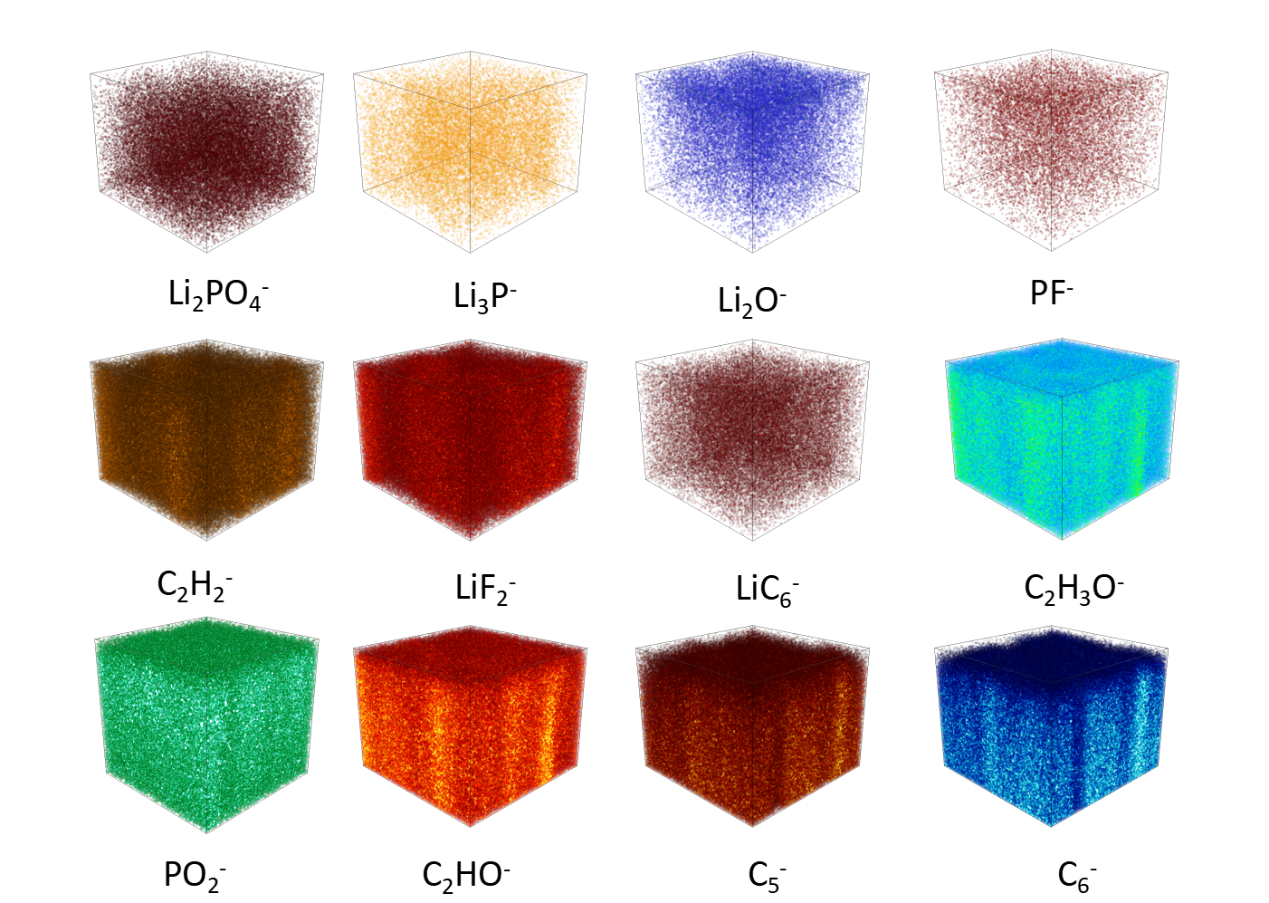


**Fig. S19.** Negative-mode TOF-SIMS depth 3D renderings profiles of Li_3_PO_4_^−^, Li_3_P^−^, Li_2_O^−^, PF^-^, C_2_H_2_^-^, LiF^-^, LiC_6_^-^, C_2_H_3_O^-^, PO_2_^-^, C_2_HO^-^, C_5_^-^ and C_6_^−^ in DES-G.

**Table S1.** The consumption of reagents by different recycling methods (kg/kg).

| Reagents | Graphene | Graphitization | Our work |
| --- | --- | --- | --- |
| Sulfuric acid | 90 | 1 | - |
| Phosphoric acid | 10 | - | - |
| Phytic acid | - | - | 1.5 |
| Choline chloride | - | - | 0.25 |
| KMnO_4_ | 4.5 | - | - |
| Hydrogen peroxide | 7.5 | 1.8 | - |
| NaOH | - | 1.5 | - |
| Product | 0.5 | 0.6 | 0.8 |

**Table S2.** Environmental and energy consumption of different recycling methods.

| Methods | Graphene | Graphitization | Our work |
| --- | --- | --- | --- |
| effluent (kg) | 1000 | 500 | 100 |
| Temperature (℃) | 0-5 | 2500-3000 | 80 |
| power (kW) | 0.2 | 100 | 1.5 |
| energy consumption (MJ/Kg) | 17 | 180 | 21 |
| CO_2_ emission (Kg) | 6.7 | 13 | 0.5 |

**Table S3.** Economic analysis of different recycling methods ($/kg).

| Methods | Graphene | Graphitization | Our work |
| --- | --- | --- | --- |
| Revenue | 1400 | 4.20 | 5.70 |
| Cost | 1521 | 2.57 | 2.05 |
| Profit | −121 | 1.63 | 3.65 |

**Table S4.** The chemical composition of waste graphite before and after DES regeneration.

| Elements | C | Li | Al | Co | Cu | Ni | Fe | Mn | F | P |
| --- | --- | --- | --- | --- | --- | --- | --- | --- | --- | --- |
| Before (ppm) | 97.6% | 873 | 537 | 261 | 673 | 682 | 1064 | 326 | 2041 | 932 |
| After (ppm) | 98.4% | 0 | 25 | 0 | 0 | 0 | 12 | 6 | 65 | 1.5% |
